# Plasma and Tissue Specific miRNA Expression Pattern and Functional Analysis Associated to Colorectal Cancer Patients

**DOI:** 10.3390/cancers12040843

**Published:** 2020-03-31

**Authors:** Roxana Cojocneanu, Cornelia Braicu, Lajos Raduly, Ancuta Jurj, Oana Zanoaga, Lorand Magdo, Alexandru Irimie, Mihai-Stefan Muresan, Calin Ionescu, Mircea Grigorescu, Ioana Berindan-Neagoe

**Affiliations:** 1 Research Center for Functional Genomics and Translational Medicine, Iuliu Hatieganu University of Medicine and Pharmacy, 23 Marinescu Street, 400015 Cluj-Napoca, Romania; roxana.cojocneanu@umfcluj.ro (R.C.); raduly.lajos78@gmail.com (L.R.); anca.jurj@umfcluj.ro (A.J.); oana.zanoaga@umfcluj.ro (O.Z.); lorand.magdo@gmail.com (L.M.); ioana.neagoe@umfcluj.ro (I.B.-N.); 2 Department of Surgery, The Oncology Institute “Prof. Dr. Ion Chiricuta”, 400015 Cluj-Napoca, Romania; airimie@umfcluj.ro; 3 Department of Surgical Oncology and Gynecological Oncology, University of Medicine and Pharmacy Iuliu Hatieganu, 400015 Cluj-Napoca, Romania; 4 5th Surgical Department, Municipal Hospital, 400139 Cluj-Napoca, Romania; mihai.stefan.muresan@gmail.com; 5 Department of Surgery, Iuliu Hatieganu University of Medicine and Pharmacy, 23 Marinescu Street, 400015 Cluj-Napoca, Romania; 6 Gastroenterology and Hepatology Department, 3rd Medical Clinic, Iuliu Hatieganu University of Medicine and Pharmacy, 400162 Cluj-Napoca, Romania; mircea.grigorescu@gmail.com; 7 MEDFUTURE-Research Center for Advanced Medicine, University of Medicine and Pharmacy Iuliu Hatieganu, 23 Marinescu Street, 400015 Cluj-Napoca, Romania; 8 Department of Functional Genomics and Experimental Pathology, The Oncology Institute “Prof. Dr. Ion Chiricuta”, Republicii 34 Street, 400015 Cluj-Napoca, Romania

**Keywords:** colorectal cancer, plasma, microarray, miRNA, biomarker

## Abstract

An increasing number of studies suggest the implication of microRNAs (miRNAs) in colorectal (CRC) carcinogenesis and disease progression. Nevertheless, the basic mechanism is not yet clear. We determined plasma miRNA expression levels using Agilent microarray technology followed by overlapping with The Cancer Genome Atlas (TCGA) tissue data and a qRT-PCR validation step and analysis of the altered miRNA signatures to emphasize new mechanistic insights. For TGCA dataset, we identified 156 altered miRNAs (79 downregulated and 77 upregulated) in colorectal tissue samples versus normal tissue. The microarray experiment is based on 16 control samples, 38 CRC plasma samples from colorectal cancer patients who have not undergone chemotherapy, and 17 chemo-treated samples. In the case of the analysis of CRC cancer versus healthy control we identified 359 altered miRNAs (214 downregulated and 60 upregulated), considering as the cutoff value a fold-change of ±1.5 and *p* < 0.01. An additional microarray analysis was performed on plasma from untreated colorectal cancer (*n* = 38) and chemotherapy-treated colorectal cancer patients (*n* = 17), which revealed 15 downregulated miRNAs and 53 upregulated miRNAs, demonstrating that the plasma miRNA pattern is affected by chemotherapy and emphasizing important regulators of drug resistance mechanisms. For the validation of the microarray data, we selected a panel of 4 miRNAs from the common miRNA signatures for colon and rectal cancer (miR-642b-3p, miR-195-5p and miR-4741). At the tissue level, the expression levels were in agreement with those observed in colorectal plasma. miR-1228-3p, the top upregulated miRNA in CRC, was chosen to be validated on tissue and plasma samples, as it was demonstrated to be downregulated at tissue level in our patient cohort. This was confirmed by TCGA data and was one example of ta ranscript that has a different expression level between tumor tissue and plasma. Developing more efficient investigation methods will help explain the mechanisms responsible for miRNAs released in biofluids, which is the most upregulated transcript in colorectal plasma samples and which can function as a prediction tool within the oncological field.

## 1. Introduction

Colorectal carcinoma is the most common tumor affecting the entire digestive tract [1]. Colorectal cancer is a highly prevalent disease which, according to Globocan 2018, ranks fourth in terms of both incidence (9.7%) and mortality (8.4%) worldwide, for both genders combined [2,3,4], with more than half of patients identified in regions which present a higher degree of development. This malignancy is the second most common type of cancer to affect women (with both incidence and mortality values around 9%), right after breast and cervical cancer, and the third in men, after lung and prostate cancers (with an incidence of 10.1% and a mortality of 8%). Diagnosed in its early stages, colorectal cancer can be treated with considerable success rates [5], and this is one of the reasons why it is of utmost importance to drastically change the clinical approach to this malignancy, in terms of identifying fast, accurate, minimally invasive diagnostic and prognostic methods, as well as developing more efficient targeted therapies [4,6,7,8,9]. Therefore, there is a growing necessity for identifying and characterizing the molecular pathogenesis of colorectal carcinoma [8,10,11].

It has been shown that miRNAs have an important role in tumorigenesis, and the involvement of these transcripts in the etiology of cancer is a subject of study for researchers across the world [12,13,14,15]. miRNAs are non-coding RNA structures, of 19–24 nucleotides, which are never translated into proteins, but that have a role in the control of the expression of protein coding genes by either physically degrading the messenger RNA or by blocking transcription [13,16,17,18]. Studies have shown that miRNAs are commonly found in circulation, both in serum and plasma, either free or “encapsulated” within small vesicles named exosomes [15,19,20,21]. These short transcripts are presumably excreted by tumors and released into circulation; therefore, they carry information regarding the place of origin [22,23] and thus are able to play diagnostic and prognostic roles regarding the development and progression of tumors [13,14,21,24,25].

High throughput technologies such as microarray or next generation sequencing have allowed the evaluation of molecular profiles in various types of cancers, in an attempt to understand the complexity and heterogeneity of the malignant disease [15,21,26,27]. Considering all these, one of the main objectives of our study was to evaluate plasma miRNAs and to compare them with the expression levels found in tissue (using The Cancer Genome Atlas (TCGA) data) to identify colorectal-cancer-specific miRNAs. Understanding the regulation of miRNA expression has the potential to shed light on the screening of cancer biomarkers, particularly those related to the response to therapy, and the initiation of resistance behavior.

## 2. Material and Methods

### 2.1. TCGA Data, Human Subjects and Clinical Data

The Cancer Genome Atlas (TCGA) is a public database developed to create a comprehensive cancer genomic profile with important implications in biomarker discovery [28]. Starting from this, we designed and performed a microarray experiment for microRNA evaluation on plasma samples from colorectal cancer (CRC). In order to calculate the differential expression, colorectal samples were compared with plasma collected from healthy controls deposited in our biobank. All samples were obtained by following protocols in strict compliance with national and European regulations regarding the manipulation of biological samples and personal data: all cases were anonymized, all donors were briefed about the study and each gave informed consent. At the same time, the study and all its aspects were approved by the Ethical Committee of The Oncology Institute “Prof. Dr. Ion Chiricuta” (approval number 6346/02.07.2014), which certifies the compliance with the aforementioned regulations, including the proper signing of the informed consent forms by all participants in the study.

TCGA clinical data for the colorectal samples used in present study are presented in Table 1. The microarray experiment was conducted on a total of 55 CRC plasma samples: 38 samples without chemotherapy (CT) and 17 post-chemotherapy, together with 16 control samples. According to the anatomical site, 25 cancer cases were located on the colon, 25 on the rectum, and five cases had colorectal localization. The complete demographic characteristics of the patients are presented in Table 2, alongside some information regarding the healthy controls for the microarray study. Table 3 contains demographic characteristics for the validation set.

### 2.2. Plasma Preparation and RNA Isolation for Colorectal Cancer Patients and Healthy Controls

At the time of sample collection, peripheral blood on EDTA (Ethylenediaminetetraacetic acid) was obtained from each patient after the initial diagnosis, and immediately processed (within two hours from collection) to obtain the plasma by centrifugation at 4000 rpm for 10 minutes at room temperature, under standardized conditions. Plasma was divided into aliquots and deposited at −80 °C. The extraction of miRNAs from the plasma samples was performed using the Plasma/Serum Circulating and Exosomal RNA Purification Kit (Slurry Format). After extraction, the nucleic acids were subjected to quantitative and qualitative evaluation, using the NanoDrop instrument, and the Agilent Bioanalyzer (Agilent Technologies, Santa Clara USA), respectively. This way, we were able to ensure the necessary values for the nucleic acid concentrations that are mandatory for the subsequent experimental steps.

### 2.3. miRNA Microarray Evaluation

The protocol for the miRNA microarray experiment started with achieving a concentration of 100 ng of total RNA for each of the studied samples. Sample hybridization was performed using the Agilent microRNA Spike-In kit, while labeling was done with the miRNA Complete Labeling and Hyb Kit. In order to avoid the possible presence of artifacts, we preceded with a purification step, with the help of Micro Bio-Spin 6 (Biorad, Mississauga, ON, Canada) spin columns, followed by a desiccation step in a vacuum centrifuge, and the resuspension of the pellet in 18 μL of RNase free, microbiologically pure water. The hybridization phase was conducted according to the manufacturer’s recommendations, and the slides (Agilent SurePrint Human miRNA v21.0 microarray, G4872A) were left in the hybridization oven for 20 hours, at a temperature of 55 °C. After washing, the slides were scanned using an Agilent Microarray Scanner.

### 2.4. Analysis of Microarray Data

After scanning the microarray slides, the Agilent Feature Extraction software (Agilent Technologies) was used to analyze the images and convert them to numeric expression values. The individual files were fed into the Agilent GeneSpring GX program (Agilent Technologies) for data normalization, which was conducted using the quantile algorithm. No baseline transformation was performed. Entities were initially filtered based on their flag values, keeping them acceptable on the “detected” ones. Differentially expressed genes were selected using the “Filter on Volcano Plot” analysis and moderated t-test, with a fold change of 1.5 and a *p*-value <0.05 for the following comparisons: treated samples versus untreated samples, untreated colon samples versus controls, untreated rectum samples versus controls, and treated rectum samples versus untreated rectum samples. The missing logical comparison “treated colon samples versus untreated colon samples” is due to the fact that the cohort of patients did not include any patients with colon localization of tumors who previously underwent chemotherapy. Whenever possible, the Benjamini–Hochberg (false discovery rate (FDR)) algorithm was applied, to control for false positives.

### 2.5. miRNA RT-PCR

For testing the candidate miRNAs (miR-1228-3p, miR-4741, miR-642b-3p, miR-195-5p) observed to be differentially expressed in a statistically significant manner in the microarray experiment, RT-PCR was performed starting from 50 ng total RNA. The total RNA was reverse transcribed using a TaqMan MicroRNA Reverse Transcription Kit (Applied Biosystem, 4366596). RT-PCR reactions were performed on the ViiA7 instrument (Applied Biosystems, Foster City, CA, USA) in a 10μL reaction volume containing TaqMan™ Fast Advanced Master Mix (Catalog number: 4444556, Thermo Scientific, Waltham, MA, USA). The expression level was calculated using the 2^−ΔΔCt^ method, using U6 for normalization and *p* < 0.05 was considered statistically significant. A ROC (receiver operating characteristic) analysis was employed to calculate sensitivity and specificity of each biomarker using GraphPad Prism (https://www.graphpad.com/, Version 6). 

### 2.6. Integrated Analysis of the Altered Plasma miRNAs in Colorectal Cancer

The integrated analysis for the altered miRNA transcripts was performed using Ingenuity Pathway Analysis (IPA; Qiagen, Inc., Valencia, CA, USA), which is a valuable bioinformatics tool used for the identification of the most relevant altered pathways and networks based on the complex database with 302 metabolic networks and 360 signaling pathways. The altered miRNAs are classified based on a significance score by overlapping with these networks and pathways. For the integration of the altered plasma miRNA patterns in drug resistance mechanisms, the NCBI Gene database was used (www.ncbi.nlm.nih.gov) by searching for the string “miRNA” and “drug resistance” and by selecting the non-coding genes for *Homo sapiens*. For evaluating the target genes we used miRTargetLink database (https://ccb-web.cs.uni-saarland.de/mirtargetlink/). 

## 3. Results 

### 3.1. Differential miRNA Expression on Colorectal Cancer Based on TCGA Data

Using as cutoff values a fold-change (FC) of ± 1.5 and *p* < 0.05, in the case of the TCGA analysis for colon tumor tissue versus normal tissue we obtained 276 altered miRNAs (142 downregulated and 134 upregulated). TCGA analysis for rectal tumor tissue versus normal tissue revealed 239 altered miRNAs (112 downregulated and 127 upregulated), while the global analysis comprising all the TCGA colorectal tumor tissue versus normal tissue generated 156 altered miRNAs (79 downregulated and 77 upregulated). The complete list of the altered miRNAs is presented in Tables S1–S3.

### 3.2. Differential miRNA Expression in Colorectal Plasma Samples

**Figure 1:**
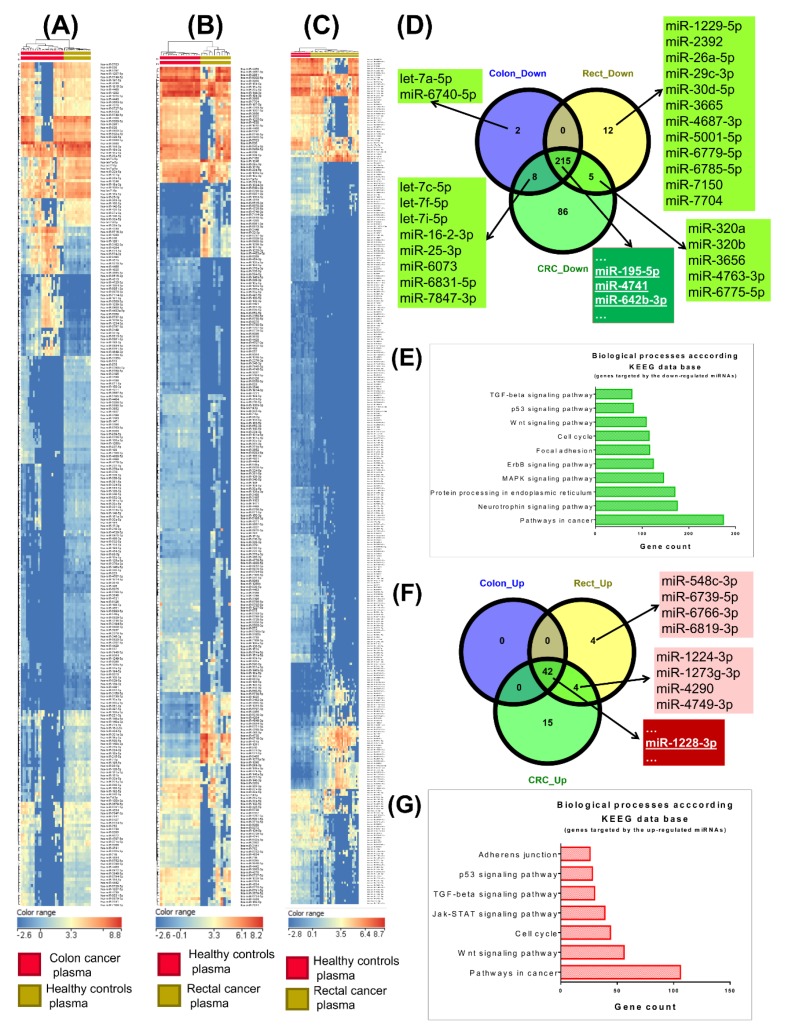
Summary of common miRNAs which are differentially expressed in plasma samples in colon, rectal, and colorectal cancer, respectively, versus healthy controls, displayed as heatmap and Venn diagrams. (**A**–**C**) Heatmap of miRNA microarray expression data for (**A**) colon, (**B**) rectal, (**C**) colorectal cancer versus healthy subjects. (**D**) The common miRNAs are presented by overlapping downregulated miRNAs in the three analyzed groups. (**E**) Biological processes affected, identification of target genes for the downregulated miRNAs using miRnet connected with KEEG database. **(F**) The common miRNAs are presented by overlapping overexpressed miRNAs in the three analyzed groups. (**G**) Biological processes affected, identification of target genes for the upregulated miRNAs using miRnet connected with KEEG database.

**Figure 2:**
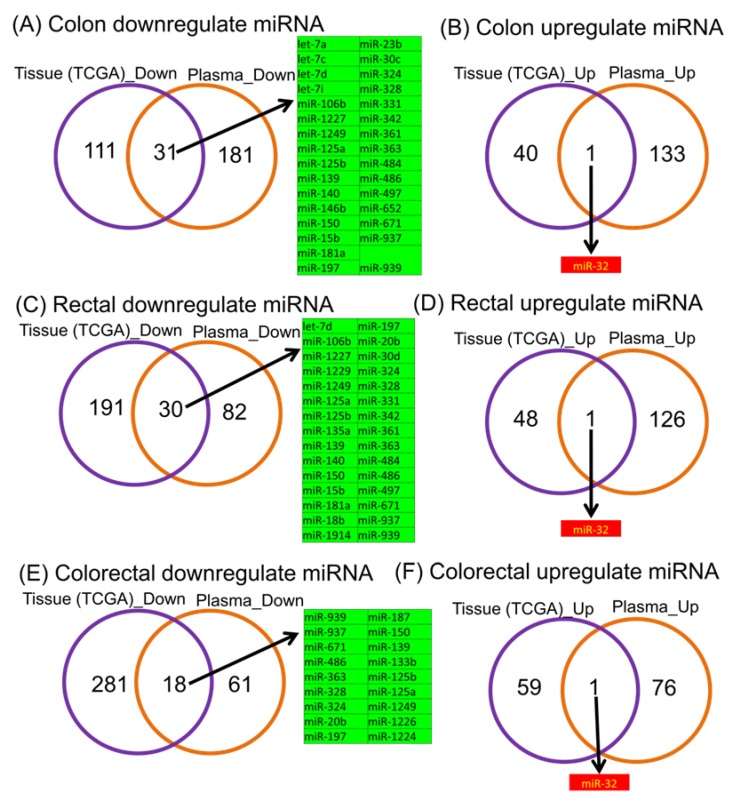
Common miRNA expression profile pattern in TCGA (tumor versus normal tissue) and plasma (tumor samples versus healthy controls) data on colon, rectal and colorectal cancer. The diagrams present the overlapping TCGA and plasma miRNA signatures in the case of upregulated (**A**) and downregulated transcripts (**B**) for the case of colon cancer, similarly for the case of downregulated (**C**) and overexpressed miRNAs (**D**). We also present the overlapping of the downregulated (**E**) and overexpressed miRNAs (**F**) transcripts for colon, rectal and colorectal cancer.

The microarray experiment is based on 38 plasma samples from colorectal cancer patients who have not undergone chemotherapy, and 16 control samples. Using a FC cutoff value of ± 1.5 and *p* < 0.05, the differential expression analysis of colon cancer plasma samples versus healthy controls revealed 255 altered miRNAs (214 downregulated and 41 upregulated), while in the case of rectal cancer plasma samples versus healthy controls we observed 270 altered miRNAs (221 downregulated and 49 upregulated). The global analysis comprising colorectal plasma samples versus healthy controls revealed 359 altered miRNAs (299 downregulated and 60 upregulated). The top 15 most upregulated and downregulated microRNAs for each comparison are presented in Table 4, and the complete list in Tables S4–S6; we also show the heatmaps (Figure 1A–C) and the Venn diagrams for the downregulated miRNAs (Figure 1D) and classification of biological processes (Figure 1E). We present similar information in Figure 1F for overexpressed transcripts in the analyzed groups, and in Figure 1G the classification for biological processes; the overlapping of data from TGCA with those obtained from the plasma is displayed in Figure 2, as a Venn diagram between the analyzed groups, emphasizing the common miRNA signatures between tissue and plasma (Figure 2A,B for colon cancer, Figure 2C,D for rectal cancer, Figure 2E,F for colorectal cancer). 

### 3.3. Plasma miRNA Pattern Affected by Chemotherapy

**Figure 3:**
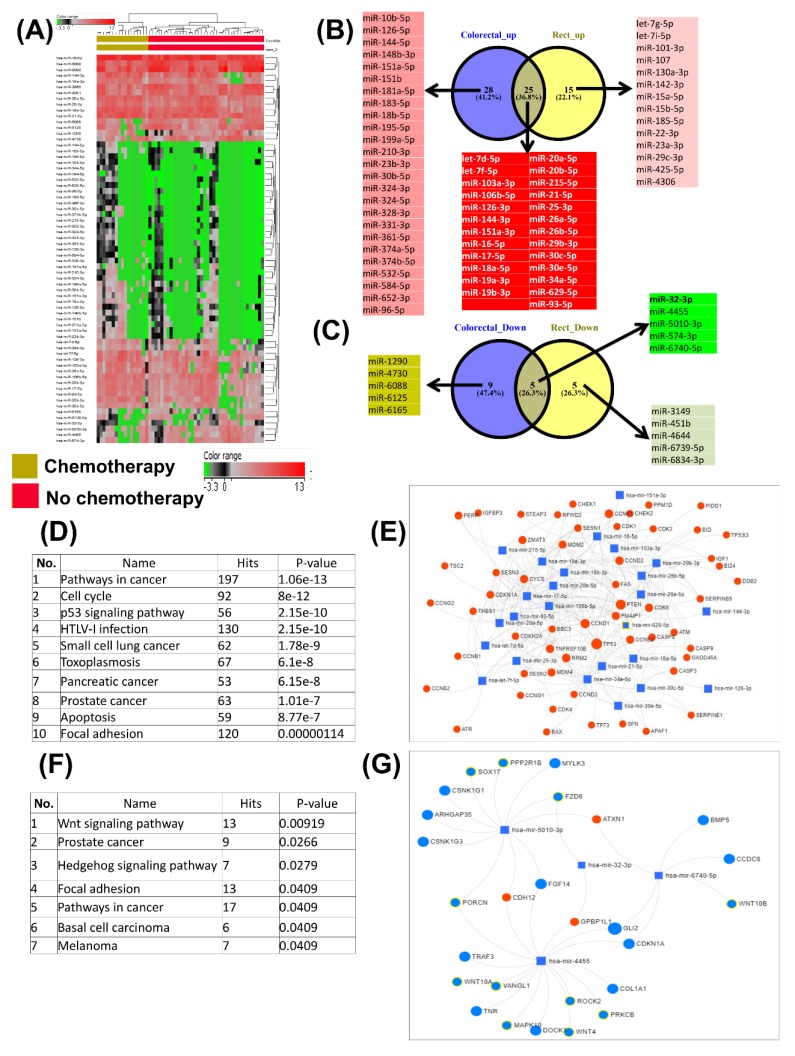
miRNA profile and pathway analysis affected by chemotherapy. (**A**) Heatmap detailing miRNA expression patterns from chemo-treated versus no chemotherapy plasma samples; (**B**) Venn diagram for the overexpressed miRNAs in colorectal and rectal cancer, emphasizing the common and specific signatures; (**C**) Venn diagram for the downregulated miRNAs in colorectal and rectal cancer; (**D**) cellular processes related to the target genes of the 25 most common overexpressed miRNA signatures in colorectal and rectal cancer. miRNA–mRNA integration was generated using miRNET and KEGG (Kyoto Encyclopedia of Genes and Genomes) classification of the target genes, based on statistical significance and strength of association; (**E**) miRNA–mRNA integration related to TP53 signaling; (**F**) cellular processes related to the target genes of the five most common downregulated miRNA signatures in colorectal and rectal cancer. (**G**) miRNA–mRNA integration was generated using miRNET and KEGG classification of the target genes based on statistical significance and strength of association.

Microarray evaluation of microRNAs in plasma from untreated colorectal cancer and chemotherapy-treated colorectal cancer patients revealed 14 downregulated miRNAs and 49 upregulated miRNAs (Table S7), data which are presented in the form of a heatmap in Figure 3A; a similar analysis for rectal cancer patients treated with chemotherapy versus untreated patients revealed ten downregulated miRNAs and 40 upregulated miRNAs, presented using a Venn diagram (Figure 3B,C). 

The five most common downregulated miRNAs were proved to target 509 genes involved in key cellular processes presented in Figure 3D, sorted based on *p*-value, while Figure 3E shows mRNA–miRNA networks, targeting genes involved in pathways in cancer, the Wnt signaling pathway and focal adhesion. The most common 25 overexpressed miRNAs target 6993 genes involved in key cellular processes, presented in Figure 3F and arranged based on *p*-value, while Figure 3G shows the miRNA network involved in p53 signaling.

### 3.4. RT-PCR Tissue and Plasma Validation

**Figure 4:**
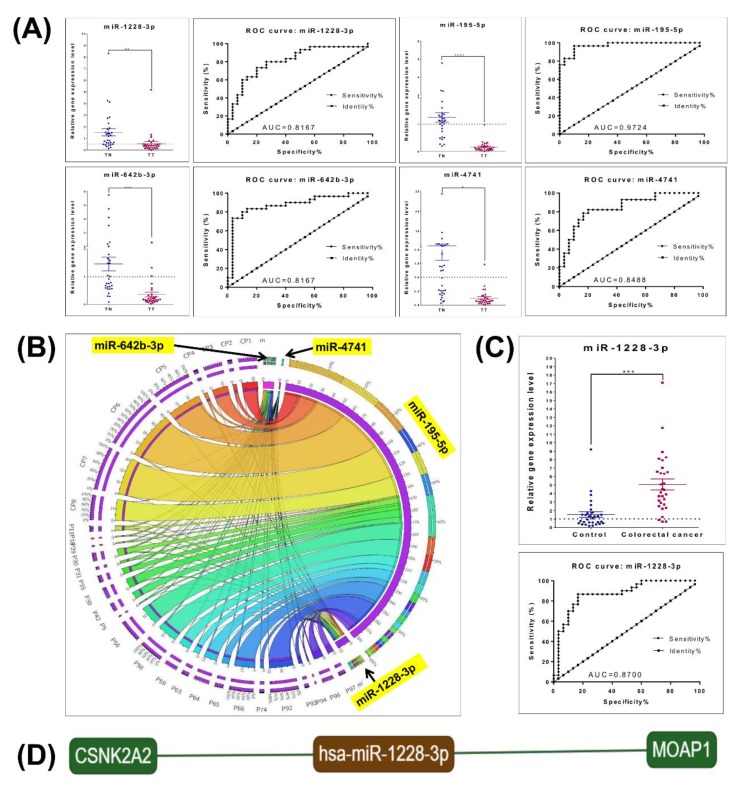
Tissue and plasma qRT-PCR data validation, the evaluation of the miRNA expression level shows relevant differences. (**A**) Expression level of miR-1228-3p, miR-642b-3p, miR-195-5p and miR-4741 in tumor tissue and normal tissue, and receiver operating characteristic (ROC) curve; (**B**) Circos plot of the integrated miRNA spatial signature showing the heterogeneity of the expression level; (**C**) miR-1228-3p expression level in plasma colorectal patients and healthy controls and ROC curve; (**D**) miR-1228-3p direct target, generated using miRtargetlink; (* *p* ≤ 0.05, ** *p* ≤ 0.01, *** *p* ≤ 0.001, **** *p* ≤ 0.0001).

For the validation of the microarray data we selected a panel of four miRNAs (miR-1228-3p, miR-642b-3p, miR-195-5p and miR-4741) that were used for validation at tissue level in an independent patient cohort (30 matched paired samples), while from the top upregulated miRNAs, miR-1228-3p was chosen to be validated in another independent plasma sample set (24 colorectal plasma samples and 25 healthy controls). The expression levels in tumor tissue for miR-642b-3p, miR-195-5p and miR-4741 is in agreement with those observed in the microarray experiment (Figure 4A), data being integrated as Circos plot (Figure 4B). miR-1228-3p is a transcript that was the most upregulated in colon, rectal and colorectal plasma microarray analyses, a fact also confirmed by qRT-PCR at plasma level; however, in tissue the expression level was downregulated (Figure 4C), the same as for the TCGA data. The direct targets for miR1228-5p are presented in Figure 4D.

**Figure 5:**
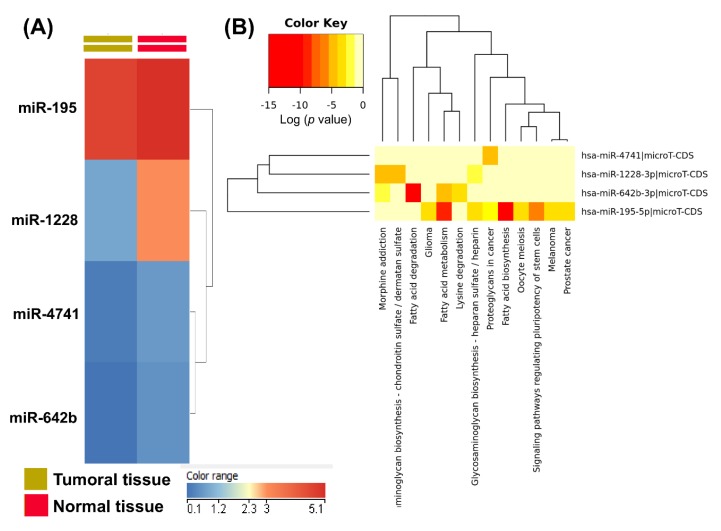
Common miRNA signatures for colon, rectal and colorectal cancers involved in drug resistance. (**A**) Heatmap of the four validated miRNAs. (**B**) Heatmap generated using DIANA-miRPath v3.0 (http://snf-515788.vm.okeanos.grnet.gr), showing miRNA targets associated with biological processes related to the validated miRNAs.

In Figure 5A is presented the heatmap for the miR-195, miR-1228, miR-4741 and miR642 based on TCGA data, meanwhile in Figure 5B is presented the heatmap representation of the significant pathways induced by the miRNA signatures obtained by KEGG pathway enrichment analysis using the DIANA Tools miRPath instrument; this demonstrated significant functional activity, particularly for the case of miR-195-5p.

### 3.5. Plasma miRNAs as Primordial Signaling Molecules in Colorectal Cancer, Evaluated Using IPA

**Figure 6:**
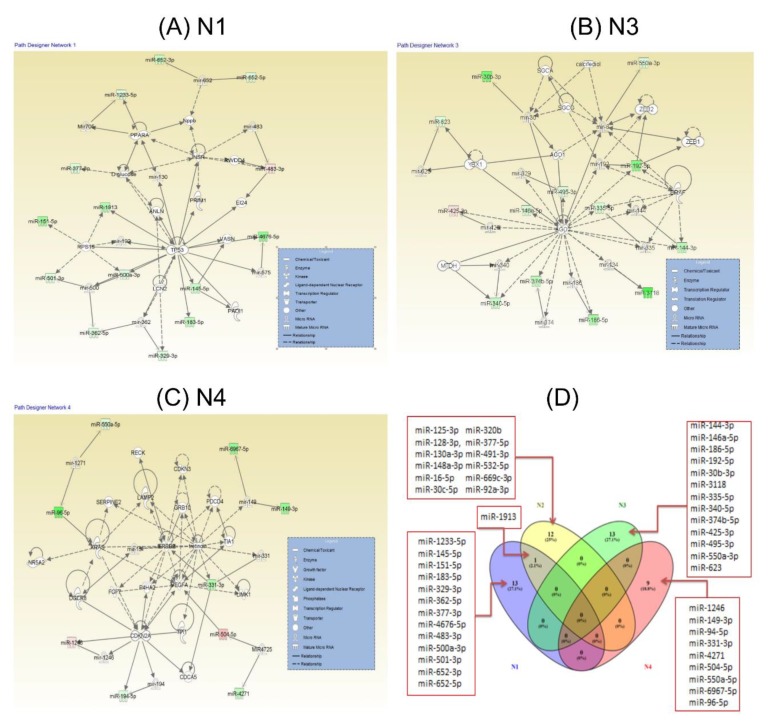
miRNA networks generated using IPA (Ingenuity Pathway Analysis) for the altered plasma miRNAs in colorectal cancer. (**A**) **N**1: Inflammatory Disease, Inflammatory Response, Organismal Injury and Abnormalities. (**B**) **N**3: Cancer, Organismal Injury and Abnormalities, Reproductive System Disease. (**C**) **N**4: Cancer, Immunological Disease, Organismal Injury and Abnormalities. (**D**) Venn diagram for miRNA altered networks.

We applied IPA (Ingenuity Pathway Analysis) to visualize potential interaction networks for the differentially expressed miRNAs between colorectal plasma versus healthy controls. This bioinformatics tool allows us to perform an integrated approach for the identification of the interconnections between the altered miRNAs and the most relevant genes. The IPA analysis is used to classify the altered miRNA transcripts in terms of the most relevant associated networks, and are summarized in Table 5 and visualized in Figure 6 the main altered networks (N1: Figure 6A, N2: Figure 6B, N3: Figure 6C and N4: Figure 6D).

### 3.6. Plasma miRNAs as Regulators for Drug Resistance Mechanisms

**Figure 7:**
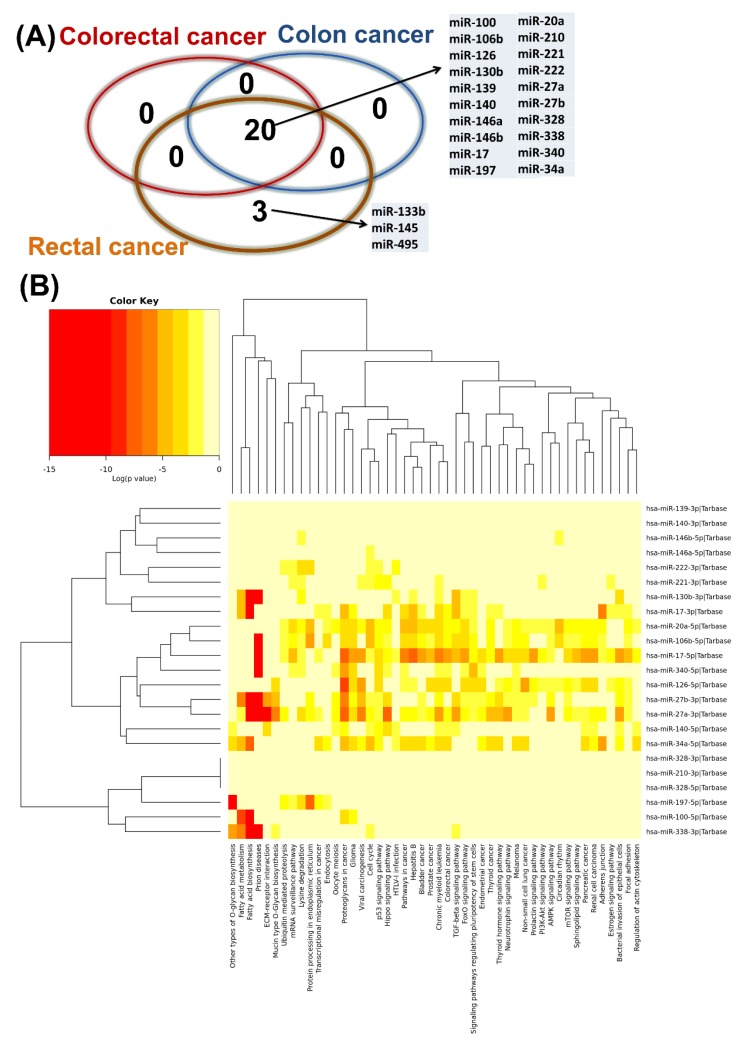
Plasma miRNAs as key modulators of drug resistance, with altered expression in colon and rectal cancer, as well as for the global analysis comprising all colorectal cancers. (**A**) Venn diagram for the altered miRNAs which supposedly interfere with drug resistance in the analyzed groups; (**B**) heatmap generated using DIANA-miRPath v3.0, showing miRNA targets associated with biological processes related to the common miRNA signatures involved in drug resistance.

Our current research revealed a higher number of altered miRNAs observed in the literature as key target genes for chemoresistance and biomarkers for treatment response. These are displayed as a Venn diagram by overlapping the NCBI miRNA list related to drug resistance with the altered miRNA patterns (Figure 7A). We identified 20 transcripts involved in drug resistance in colorectal and colon cancer, and three transcripts specific for rectal cancer. Figure 7B presents the heatmap for the most common 20 miRNAs generated using DIANA-miRPath v3.0, and shows the main biological processes involved.

**Figure 8:**
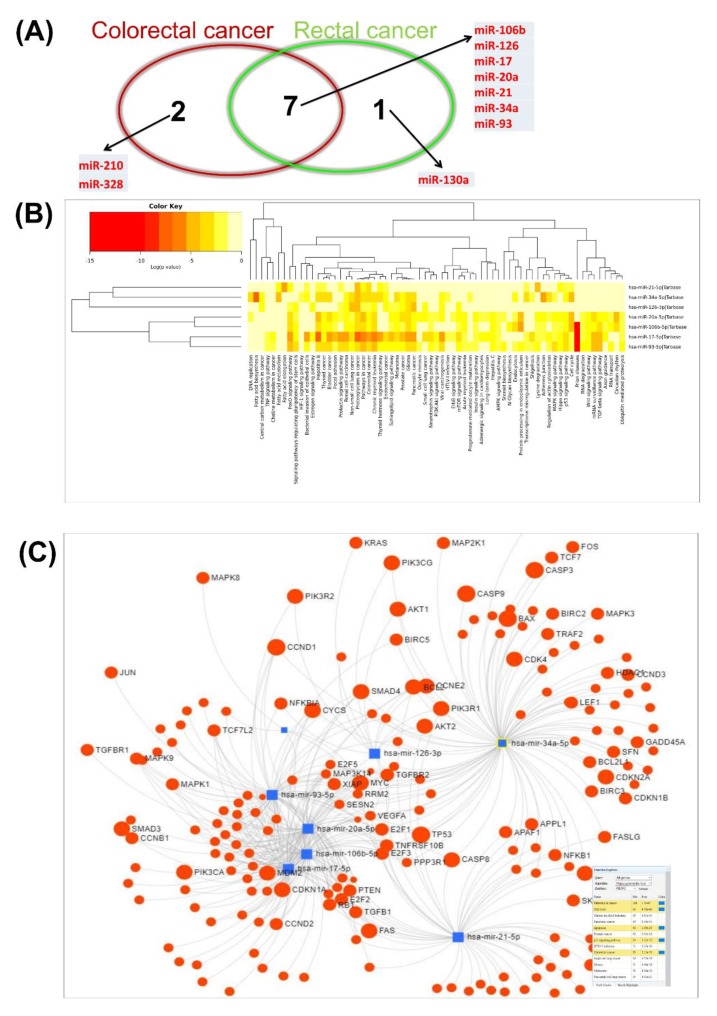
Plasma overexpressed miRNAs as key modulators of drug resistance observed to be altered in the cases of the analysis of colorectal and rectal cancer chemo-treated versus untreated. (**A**) Venn diagram for the altered miRNAs which are supposed to interfere with drug resistance in the analyzed groups. (**B**) Heatmap generated using DIANA-miRPath v3.0 (http://snf-515788.vm.okeanos.grnet.gr), showing miR-21-5p, miR-34a-3p, miR-126-3p, miR-20a-5p, miR-106-5p, miR-17-5p and miR-93-5p targets associated with biological processes. (**C**) miRNET network for common miRNA signatures emphasizing an important number of target genes related to pathways in cancer and TP53 signaling.

In the analysis for colorectal and rectal cancer chemo-treated versus untreated, we identified a panel of seven miRNAs as modulators of drug resistance mechanisms (Figure 8A). Figure 8B presents the implication of miRNA in key biological processes. The miRnet network for common miRNA signatures emphasized an important number of target genes related to pathways in cancer (*n* = 118 targets, *p*-value = 1.7 × 10^−97^) cell cycle regulation (*n* = 54 targets, *p*-value = 4.76 × 10^−43^), apoptosis (*n* = 40 targets, *p*-value = 1.08 × 10^−33^) and TP53 signaling (*n* = 118 targets, *p*-value = 3.22 × 10^−32^) (Figure 8C). 

## 4. Discussion 

Tumor-specific alterations of plasma miRNAs in cancer patients are promising non-invasive biomarkers with diagnostic/prognostic value. Plasma miRNAs have a high stability, therefore emphasizing their promising application as biomarkers [29,30]. Moreover, a better comprehension of miRNA trafficking (intracellular, extracellular realized in different forms, free or via vesicles) is essential for understanding the biology of cancer, and a particular attention should be directed to the vesicles that support the processes of exiting and entering the bloodstream circulation. miRNAs are key players known to participate in tumorigenesis and regulate the development and progression of human malignancies [13,14]. In our case, we identified differences among plasma miRNA profiles, most of them being downregulated.

A limited number of studies present miRNA profiling data for colorectal cancer at the plasma level. This is not always correlated with what we observed by the overlapping of plasma miRNA data with those from TCGA. Furthermore, we noticed a larger variation in miRNA expression levels among colon and rectal cancer plasma samples, suggesting that colon and rectal cancer should be studied independently, taking into account the molecular and clinical incongruities. Consequently, the general observation is that miRNAs are downregulated in colon and rectal cancer, with similar observations presented in a recent miRNA profiling study on rectal cancer tissue versus normal tissue [31].

Plasma-released miRNAs may be considered as a promising strategy for increased therapeutic efficacy and particularly for avoiding the activation of drug resistance mechanisms, as we observed in the case of the functional study performed on miR-205 [32]. The network presented in Figure 6A is centered on the key gene TP53, which is involved in the regulation of cell proliferation, but was also proved to interfere with drug resistance related pathways [33]. The modulation of miRNA-processing enzymes Ago2 by miRNAs (Figure 6B) may be important for the regulation of cell proliferation and EMT (epithelial to mesenchymal transition) based on the fact that they are interconnected with two main effectors of this mechanism (Zeb1 and Zeb2), which are regulated by miR-205. Ago2 and related enzymes involved in miRNA biogenesis are important effectors in colorectal carcinomas [34]. Ago1 and Ago2 are associated with selective autophagy [35], and are able to target cell cycle regulators, cell proliferation and apoptosis [36,37,38], as observed in Figure 6C.

There is growing concern for establishing correlations between miRNA expression in tumors or the alteration of expression levels as a response to chemotherapy and radiosensitivity, which are considered as predictors or modifiers of the response to therapy [39]. As presented previously, miRNAs can be used as biomarkers, and here we present a specific panel of transcripts (Figure 7) that can be considered as candidate markers for predicting the response to chemotherapy [30].

miR-1228-3p was found to be downregulated in our colorectal cancer tissue samples and TCGA dataset. Moreover, microarray and RT-PCR validation assays on plasma samples from the same patients revealed a significant overexpressed profile, a fact that could classify this miRNA as a strong prediction biomarker. The opposite expression level of miR-1228 between plasma and tissue samples is an intriguing outcome, where a possible mechanism could consist of the expulsion of this miRNA in circulation via exosomes for the further rescue of tumor cells from a tumor suppressor miRNA [40]. The two validated targets of miR-1228 identified using the miRTargetLink database might have important relevance in colorectal cancer (Figure 4E). The first gene, MOAP1, has been previously identified as an important tumor suppressor molecule linked to the RASSF1A protein, with implications in apoptosis [41]. Data from The Human Protein Atlas [42] reveal that this protein is actually downregulated in colorectal cancer patients; the fact is that, in some instances, this can be contradictable with miR-1228-3p expression (which is also downregulated in tumor tissue). However, a possible explanation could involve the methylation status, where the downregulation of MOAP1 is independent of miR-1228 regulation in colorectal cancer due to epigenetic modifications in RASSF1A [43]. We further concentrated on the second strong validated target gene of miR-1228-3p, CSNK2A2. This gene was previously found as overexpressed in colorectal cancer and was also affiliated with late stages and chemotherapeutic response (5-FU in vitro-related, HCT116 parental and chemotherapy-resistant cell line models using a disease-specific microarray) [44]. Specifically, CSNK2A2 protects colon cancer cells from TRAIL-induced apoptosis [45]. A possible explanation for this overexpression pattern in colorectal cancer could be offered by miR-1228 downregulation in tissue samples, where experimental upregulation of the miRNA could stand as a potent therapeutic strategy. With this in mind, miR-1228-3p could become an important biomarker in colorectal cancer and also a therapeutic target due to the paradoxical expression in tissue and plasma samples that most probably involves an exosomal shifting mechanism, and also due to the inhibitory roles of key genes involved in cell death pathways (with further consequences on drug resistance).

Within this framework, the research presented in this manuscript is intended to be further developed in the future, with the purpose of addressing one of its limitations, i.e., the relatively small number of patients enrolled in the study. Thus, we plan to conduct more types of experimental evaluations on larger cohorts of patients, in order to validate and strengthen the present results. 

## 5. Conclusions

Circulating miRNAs represent an important source of biomarkers for minimally invasive diagnosis and prognosis methods. More importantly, the evaluation of treatment response through circulating miRNAs becomes a competitive advantage in cancer treatment and management, where the current methods of evaluation are invasive and restricted in terms of specificity. More efforts in investigation methods will help explain the mechanisms responsible for miRNA-released biofluids, with direct effects upon their exploitation as biomarkers, using standardized methods. This is the case of miR-1224, which is the most upregulated transcript in colorectal plasma samples and which can function as a prediction tool within the oncological field. Moreover, this miRNA was found to have opposite expression levels in tumor samples from the same patients, demonstrating a complex mechanism of regulation, possibly mediated by exosomal shifting between the tumor mass and fluid microenvironment. 

Even if considerable progress was achieved in the comprehension of miRNA involvement in colorectal cancer, many queries must still be answered before transcribing miRNA profiling into clinical practice. One of the greatest questions for future years will be related to the identification of specific miRNA signatures for discrimination between different specific subtypes, which need to be highly reproducible and independently predictive of clinical–biological features of the tumor to ameliorate diagnosis and treatment. In the future, clinicians may perhaps be guided by simple tests evaluating the miRNA expression pattern in their patients, making them forcefully valid for cancer personalized medicine.

## Figures and Tables

**Table 1:** Clinical data for the The Cancer Genome Atlas (TCGA) dataset, representing 444 cases of colon adenocarcinoma (COAD) and 161 cases of rectum adenocarcinoma (READ).

Demographics		COAD (*n* = 444)	READ (*n* = 161)	Total CRC (*n* = 605)
Sex	Males	231	86	317
	Females	211	74	285
	Unknown	2	1	3
Age	Median, Range	68, 31–90	65.5, 31–90	68, 31–90
	Median, Range ♂	69, 31–90	64, 33–87	68, 31–90
	Median, Range ♀	68, 34–90	67, 31–87	68, 31–90
Relevant family history	Yes	56	15	71
	No	319	121	440
	Unknown	69	25	94
TNM	T1	11	9	20
	T2	74	27	101
	T3	301	110	411
	T4	55	13	68
	Tis	1	-	1
	T unknown	2	2	4
	N0	257	80	337
	N1	104	44	148
	N2	81	33	114
	Nx	-	2	2
	N unknown	2	2	4
	M0	320	121	441
	M1	65	23	88
	Mx	50	14	64
	M unknown	9	3	12
Tumor stage	I	73	29	102
	II	169	48	217
	III	125	50	175
	IV	65	24	89
	Unknown	12	10	22
Microsatellites	MSI high	76	4	80
	MSI low	78	19	97
	MSS	277	136	413
	Unknown	13	2	15

**Table 2:** Clinical data for the patients used for microarray study, representing 38 cases without chemotherapy (CT), 17 with CT and 16 healthy controls.

Demographics		Without CT (*n* = 38)	With CT (*n* = 17)	Controls (*n* = 16)
Sex	Males	18	12	9
	Females	20	5	7
Age	Median, Range	63, 41–77	61, 42–79	54.5, 41–63
	Median, Range ♂	60, 50–75	63, 42–79	55, 51–63
	Median, Range ♀	64, 41–77	58, 49–64	54, 41–58
Origin	Urban	26	10	16
	Rural	12	7	-
Relevant family history	Yes	5	2	-
	No	33	15	-
	Unknown	-	-	-
TNM	T1	3	1	-
	T2	6	6	-
	T3	26	10	-
	T4	3	0	-
	N0	22	12	-
	N1	7	5	-
	N2	9	0	-
	M0	33	15	-
	M1	5	2	-
	M - unknown	-	-	-
AJCC Staging	I	7	4	-
	IIA	12	6	-
	IIB	1	-	-
	III	1	-	-
	IIIA	-	3	-
	IIIB	8	2	-
	IIIC	4	-	-
	IV	5	2	-
Tumor grade	1	11	4	-
	2	24	11	-
	3	3	2	-
	Unknown	-	-	-
Tumor location	Colon	25	0	-
	Rectum	12	13	-
	Junction	1	4	-
Chemotherapy	No	38	0	-
	Yes	0	17	-

**Table 3:** Clinical data for the patients used for qRT-PCR, representing 24 colorectal plasma samples, 25 healthy controls and 30 matched paired tumor–normal tissue samples.

Clinical Features	Variable	Plasma		Tissue
		All Patients (*n* = 25)	Controls (*n* = 25)	All Patients (*n* = 30)
Sex	Males	13	17	15
	Females	12	8	15
Age	Median, Range	65, 37–86	44, 38–61	62, 19–79
	Median, Range ♂	59, 37–84	42, 40–61	58, 19–74
	Median, Range ♀	72, 40–86	46.5, 38–54	66, 56–79
Origin	Urban	20	25	21
	Rural	5	0	9
Relevant family history	Yes	1	-	6
	No	16	-	24
	Unknown	8	-	-
TNM	T1	1	-	4
	T2	0	-	3
	T3	14	-	19
	T4	10	-	4
	N0	11	-	20
	N1	10	-	4
	N2	4	-	6
	M0	14	-	26
	M1	6	-	4
	M – unknown	5	-	-
AJCC Staging	I	1	-	7
	II	1	-	1
	IIA	2	-	9
	IIB	-	-	1
	III	2	-	6
	IIIB	7	-	2
	IIIC	1	-	-
	IV	5	-	4
	Uncertain	6	-	-
Tumor grade	1	10	-	10
	2	11	-	17
	3	3	-	3
	Unknown	1	-	-
Tumor location	Colon	22	-	23
	Rectum	2	-	6
	Junction	1	-	1
Chemotherapy	No	25	-	30
	Yes	0	-	0

**Table 4:** Top 15 most upregulated and downregulated plasma microRNAs.

Colon Cancer versus Healthy Patients			Rectal Cancer versus Healthy Patients			Colorectal Cancer versus Healthy Patients		
Systematic Name	FC (abs)	*p* (Corr)	Systematic Name	FC (abs)	*p* (Corr)	Systematic Name	FC (abs)	*p* (Corr)
**miR-195-5p**	−20.10	2.23 × 10^−15^	miR-4530	−28.78	2.59 × 10^−6^	**miR-4741**	−19.07	2.57 × 10^−12^
miR-363-3p	−19.81	3.28 × 10^−7^	miR-6850-5p	−27.90	1.04 × 10^−5^	**miR-642b-3p**	−18.98	4.89 × 10^−10^
miR-96-5p	−18.73	1.14 × 10^−18^	**miR-642b-3p**	−26.12	6.43E × 10^−11^	**miR-195-5p**	−16.93	2.50 × 10^−16^
**miR-4741**	−14.42	8.34 × 10^−8^	miR-5787	−24.89	2.93 × 10^−5^	miR-134-5p	−15.94	1.57 × 10^−12^
miR-374a-5p	−13.10	1.69 × 10^−7^	miR-5703	−23.46	7.31 × 10^−5^	miR-5787	−14.36	1.37 × 10^−4^
miR-660-5p	−12.87	7.03× 10^−8^	**miR-4741**	−22.70	7.13 × 10^−11^	miR-96-5p	−14.27	1.88 × 10^−16^
miR-192-5p	−12.86	3.82 × 10^−9^	miR-642a-3p	−22.42	1.47 × 10^−14^	miR-4530	−14.15	1.03 × 10^−4^
**miR-642b-3p**	−12.62	7.01 × 10^−6^	miR-8072	−21.87	1.21 × 10^−11^	miR-6791-5p	−13.44	5.09 × 10^−6^
miR-151a-5p	−11.83	1.20 × 10^−7^	miR-134-5p	−21.86	4.62 × 10^−15^	miR-4534	−13.13	6.24 × 10^−6^
miR-301a-3p	−11.82	1.45 × 10^−9^	miR-630	−21.33	1.12× 10^−4^	miR-6850-5p	−13.03	3.22 × 10^−4^
miR-30b-5p	−11.08	3.17 × 10^−6^	miR-6791-5p	−19.38	1.73× 10^−7^	miR-6068	−13.01	2.25 × 10^−7^
miR-134-5p	−10.93	8.58 × 10^−7^	miR-3663-3p	−18.29	5.84 × 10^−6^	miR-6728-5p	−12.91	2.24 × 10^−13^
miR-18a-5p	−10.83	2.55 × 10^−8^	miR-4534	−18.28	1.64 × 10^−6^	miR-363-3p	−12.63	7.60 × 10^−6^
miR-126-5p	−10.81	5.37 × 10^−9^	miR-6728-5p	−18.07	2.15 × 10^−18^	miR-642a-3p	−11.93	7.58 × 10^−4^
miR-425-5p	−10.58	5.28 × 10^−5^	miR-1227-5p	−17.65	2.65 × 10^−14^	miR-8072	−11.71	1.13 × 10^−6^
**miR-1228-3p**	27.70	6.85 × 10^−9^	**miR-1228-3p**	38.80	1.22 × 10^−11^	**miR-1228-3p**	33.80	1.03 × 10^−13^
miR-4730	25.41	1.61 × 10^−12^	miR-1238-3p	36.44	2.04 × 10^−11^	miR-1238-3p	30.58	6.37 × 10^−13^
miR-6716-3p	24.99	6.11 × 10^−12^	miR-6508-5p	35.56	4.22 × 10^−10^	miR-6069	30.11	2.96 × 10^−13^
miR-6069	24.44	1.15 × 10^−8^	miR-6737-3p	34.81	4.06 × 10^−12^	miR-6800-3p	29.83	2.08 × 10^−11^
miR-6800-3p	24.09	6.46 × 10^−8^	miR-6069	34.58	4.03 × 10^−11^	miR-6508-5p	29.39	1.6 0 × 10^−11^
miR-1238-3p	23.90	2.31 × 10^−8^	miR-1234-3p	34.11	7.48 × 10^−13^	miR-6737-3p	28.55	5.39 × 10^−14^
miR-6737-3p	21.77	7.00 × 10^−9^	miR-6800-3p	34.04	9.46 × 10^−10^	miR-6716-3p	27.72	3.38 × 10^−17^
miR-6508-5p	21.64	9.66 × 10^−8^	miR-191-3p	33.43	2.20 × 10^−11^	miR-4730	25.70	5.06 × 10^−14^
miR-451b	20.37	1.71 × 10^−7^	miR-6716-3p	28.94	1.16 × 10^−12^	miR-191-3p	24.76	1.06 × 10^−11^
miR-5010-3p	19.00	1.32 × 10^−7^	miR-3162-3p	27.82	5.99 × 10^−17^	miR-451b	24.16	1.81 × 10^−10^
miR-4433a-5p	17.96	7.84 × 10^−9^	miR-4433a-5p	27.76	2.32 × 10^−11^	miR-1234-3p	24.01	9.15 × 10^−12^
miR-191-3p	17.01	2.31 × 10^−7^	miR-6797-3p	27.48	1.92 × 10^−13^	miR-4433a-5p	23.76	1.55 × 10^−13^
miR-3162-3p	16.21	3.08 × 10^−9^	miR-1281	27.26	7.17 × 10^−14^	miR-316-2-3p	21.95	1.58 × 10^−16^
miR-1234-3p	15.44	1.04 × 10^−6^	miR-451b	25.66	1.06 × 10^−8^	miR-6797-3p	20.71	2.22 × 10^−13^
miR-1281	14.80	1.69 × 10^−7^	miR-1825	25.28	1.43 × 10^−13^	miR-1281	20.66	3.25 × 10^−13^

bold letters were used for miRNAs selected for qRT-PCR validation.

**Table 5:** The most relevant networks associated with the altered plasma miRNA patterns in colorectal versus healthy controls.

ID	Associated Network Functions	Score	Focus Molecules	*n*
1	Inflammatory Disease, Inflammatory Response, Organismal Injury and Abnormalities	21	14	N1
2	Organismal Injury and Abnormalities, Reproductive System Disease, Inflammatory Disease	20	13	N2
3	Cancer, Organismal Injury and Abnormalities, Reproductive System Disease	20	13	N3
4	Cancer, Immunological Disease, Organismal Injury and Abnormalities	13	9	N4
5	Organismal Injury and Abnormalities, Reproductive System Disease, Developmental Disorder	7	6	N5

## Supplementary Materials

The following are available online at https://www.mdpi.com/2072-6694/12/4/843/s1; Table S1: Differentially expressed miRNAs were analyzed in COAD versus normal tissue based on TCGA data. The threshold value for upregulated and downregulated genes was a fold change ≥1.5 and *p*-value ≤0.05, Table S2: Differentially expressed miRNAs were analyzed in READ cancer versus normal tissue based on TCGA data. The threshold value for upregulated and downregulated genes was a fold change ≥1.5 and *p*-value ≤0.05, Table S3: Differentially expressed miRNAs were analyzed in CRC cancer versus normal tissue based on TCGA data. The threshold value for upregulated and downregulated genes was a fold change ≥1.5 and *p*-value ≤0.05, Table S4: Differentially expressed miRNAs were analyzed in plasma colon cancer patients versus healthy controls. The threshold value for upregulated and downregulated genes was a fold change ≥1.5 and *p*-value ≤0.05, Table S5: Differentially expressed miRNAs were analyzed in plasma rectal cancer patients versus healthy controls. The threshold value for upregulated and downregulated genes was a fold change ≥1.5 and *p*-value ≤0.05, Table S6, Differentially expressed miRNAs were analyzed in plasma colorectal cancer patients versus healthy controls. The threshold value for upregulated and downregulated genes was a fold change ≥1.5 and *p*-value ≤0.05, Table S7: Differentially expressed miRNAs were analyzed in plasma chemo-treated colorectal cancer patients versus untreated colorectal cancer patients. The threshold value for upregulated and downregulated genes was a fold change ≥1.5 and *p*-value ≤0.05, Table S8: Differentially expressed miRNAs were analyzed in plasma chemo-treated rectal cancer patients versus untreated rectal cancer patients. The threshold value for upregulated and downregulated genes was a fold change ≥1.5 and *p*-value ≤0.05.

## Abbreviations

COAD	Colon adenocarcinoma
CRC	colorectal cancer
CT	Chemotherapy
KEGG	Kyoto Encyclopedia of Genes and Genomes
EDTA	Ethylenediaminetetraacetic acid
EMT	epithelial to mesenchymal transition
FC	fold-change
FDR	false discovery rate
IPA	Ingenuity Pathway Analysis
miRNA	microRNA
READ	Rectum adenocarcinoma
ROC	Receiver operating characteristic
