# Supplementary material for: Plasma and Tissue Specific miRNA Expression Pattern and Functional Analysis Associated to Colorectal Cancer Patients
Source: Cancers (Basel). 2020 Mar 31;12(4):843. doi: 10.3390/cancers12040843 (PMC7226631; doi:10.3390/cancers12040843)
Supplement: Supplementary file 1 [file cancers-12-00843-s001.pdf]

Supplementary Materials

Plasma and Tissue Specific miRNA Expression Pattern and Functional Analysis Associated to Colorectal Cancer Patients

Roxana Cojocneanu, Cornelia Braicu, Lajos Raduly, Ancuta Jurj, Oana Zanoaga, Lorand Magdo, Alexandru Irimie, Mihai-Stefan Muresan, Calin Ionescu, Mircea Grigorescu and Ioana Berindan-Neagoe

Table S1. Differentially expressed miRNAs were analyzed in COAD versus normal tissue based on TCGA data. The threshold value for upregulated and downregulated genes was a fold change ≥1.5 and *p*-value ≤0.05.

Systematic Name	FC (abs)	Regulation	*p*
hsa-mir-486	-69.17818	down	2.20E-34
hsa-mir-139	-42.967876	down	0
hsa-mir-328	-41.69208	down	0
hsa-mir-197	-31.175646	down	0
hsa-mir-129-1	-25.215168	down	8.51E-42
hsa-mir-133a-1	-23.520296	down	6.88E-17
hsa-mir-1224	-21.101189	down	1.32E-30
hsa-mir-642	-20.974354	down	1.95E-24
hsa-mir-1976	-20.189072	down	1.40E-45
hsa-mir-149	-20.158945	down	6.87E-25
hsa-mir-766	-19.219688	down	8.40E-45
hsa-mir-504	-18.5475	down	0
hsa-mir-150	-18.411493	down	3.74E-19
hsa-mir-1180	-17.228813	down	3.06E-23
hsa-mir-125a	-17.013721	down	0
hsa-mir-1306	-16.964342	down	3.71E-39
hsa-mir-145	-15.507499	down	1.48E-23
hsa-mir-490	-15.239713	down	2.56E-12
hsa-mir-574	-15.110625	down	4.45E-33
hsa-let-7d	-12.95204	down	0
hsa-mir-375	-12.896943	down	1.35E-13
hsa-mir-193a	-11.601966	down	1.18E-25
hsa-let-7b	-11.516078	down	0
hsa-mir-181a-1	-11.288839	down	2.36E-33
hsa-mir-92b	-10.94404	down	6.58E-23
hsa-mir-1296	-10.22103	down	8.94E-23
hsa-mir-378	-9.980089	down	7.81E-25
hsa-mir-193b	-9.6425	down	4.98E-22
hsa-mir-99b	-9.268363	down	9.06E-31
hsa-mir-125b-1	-9.176319	down	9.88E-16
hsa-mir-423	-9.1388445	down	1.06E-37
hsa-mir-1226	-8.494278	down	8.18E-29
hsa-let-7c	-8.370713	down	3.69E-12
hsa-mir-296	-8.16921	down	2.14E-10
hsa-mir-326	-8.118997	down	2.20E-17
hsa-mir-485	-8.062454	down	1.74E-22
hsa-mir-2110	-7.7612824	down	3.38E-26
hsa-mir-874	-7.568354	down	1.14E-15
hsa-mir-433	-7.1413856	down	9.05E-28
hsa-mir-1307	-7.0567975	down	4.28E-17
hsa-mir-484	-6.93172	down	2.52E-23
hsa-mir-671	-6.5911164	down	2.17E-26
hsa-mir-324	-6.4117045	down	1.08E-21
hsa-mir-205	-6.3746424	down	4.97E-05

hsa-mir-1249	-6.288611	down	5.85E-25
hsa-mir-1468	-6.1595893	down	1.88E-16
hsa-mir-605	-6.03544	down	3.24E-35
hsa-mir-15b	-5.841153	down	2.72E-19
hsa-mir-767	-5.7812295	down	5.37E-05
hsa-mir-589	-5.7689524	down	8.01E-27
hsa-mir-744	-5.6886044	down	8.23E-14
hsa-mir-187	-5.6664586	down	1.49E-10
hsa-mir-432	-5.5770664	down	4.11E-15
hsa-mir-92a-2	-5.5019746	down	5.26E-13
hsa-mir-1826	-5.4880443	down	5.19E-04
hsa-mir-937	-5.3118467	down	6.06E-12
hsa-mir-1266	-5.041714	down	6.02E-08
hsa-mir-885	-5.0058317	down	0
hsa-mir-133b	-4.928623	down	3.87E-06
hsa-mir-140	-4.795257	down	1.53E-20
hsa-mir-483	-4.780864	down	0.018092668
hsa-mir-132	-4.7191024	down	1.19E-18
hsa-mir-1228	-4.71424	down	4.51E-22
hsa-mir-339	-4.669636	down	5.31E-10
hsa-mir-200c	-4.651522	down	1.05E-11
hsa-mir-615	-4.6386333	down	4.06E-08
hsa-mir-652	-4.508768	down	5.19E-15
hsa-mir-342	-4.4253483	down	2.97E-10
hsa-mir-760	-4.4200187	down	7.35E-14
hsa-mir-370	-4.247514	down	2.53E-13
hsa-mir-501	-4.187298	down	3.74E-09
hsa-mir-320a	-4.0551715	down	2.17E-07
hsa-mir-487a	-3.9696276	down	2.04E-16
hsa-mir-887	-3.915102	down	7.15E-14
hsa-mir-431	-3.6844723	down	1.51E-10
hsa-mir-219-1	-3.680956	down	3.46E-11
hsa-mir-1538	-3.6361578	down	4.03E-34
hsa-mir-323	-3.6258848	down	3.86E-09
hsa-mir-361	-3.5730257	down	1.75E-12
hsa-mir-331	-3.5566528	down	6.50E-08
hsa-mir-204	-3.4982772	down	7.58E-04
hsa-mir-505	-3.3903127	down	1.92E-10
hsa-mir-877	-3.318865	down	2.23E-07
hsa-mir-1229	-3.2131255	down	3.09E-15
hsa-mir-127	-3.0669692	down	1.83E-09
hsa-let-7a-1	-3.065293	down	5.75E-11
hsa-mir-935	-3.0461838	down	0.00287151
hsa-mir-1254	-3.0458095	down	1.06E-13
hsa-mir-891a	-3.0390332	down	3.27E-06
hsa-mir-218-1	-3.0222836	down	2.19E-22
hsa-mir-1301	-3.018671	down	6.48E-08
hsa-mir-886	-2.9895837	down	2.85E-04
hsa-mir-363	-2.942934	down	1.41E-04
hsa-mir-1975	-2.906888	down	0.001363733
hsa-mir-365-1	-2.8796299	down	7.53E-07
hsa-mir-1295	-2.8428178	down	1.19E-23
hsa-mir-320c-1	-2.7553587	down	5.63E-17
hsa-mir-1271	-2.7515128	down	3.35E-06
hsa-mir-28	-2.7353003	down	3.07E-20
hsa-mir-181b-1	-2.6932461	down	9.31E-07
hsa-mir-664	-2.665683	down	4.86E-08
hsa-mir-330	-2.6462743	down	2.39E-05
hsa-mir-455	-2.6429012	down	0.002250523
hsa-mir-1275	-2.6178226	down	0.001932895
hsa-mir-1274b	-2.5617502	down	0.003962798
hsa-mir-1248	-2.5306215	down	9.26E-05
hsa-mir-30d	-2.5143723	down	4.86E-08
hsa-mir-625	-2.4814641	down	0.001394254
hsa-mir-1227	-2.471171	down	6.12E-25

hsa-mir-346	-2.462659	down	2.14E-35
hsa-let-7e	-2.4142275	down	3.02E-05
hsa-mir-500	-2.3832695	down	8.10E-05
hsa-mir-1287	-2.380573	down	4.03E-07
hsa-mir-1270	-2.3523426	down	5.28E-10
hsa-mir-214	-2.3501306	down	3.48E-04
hsa-mir-191	-2.2996209	down	1.32E-05
hsa-mir-1292	-2.2644854	down	2.80E-09
hsa-mir-940	-2.176189	down	0.00138487
hsa-mir-939	-2.1097183	down	4.76E-06
hsa-mir-320b-2	-2.0855532	down	0.004718452
hsa-mir-497	-2.062978	down	5.21E-04
hsa-mir-409	-2.0508657	down	1.45E-04
hsa-mir-632	-1.9552885	down	1.61E-07
hsa-mir-663b	-1.9491395	down	0.006409941
hsa-mir-658	-1.8801347	down	7.12E-08
hsa-mir-30c-2	-1.8182406	down	0.001374953
hsa-mir-550-2	-1.7813472	down	0.00847958
hsa-mir-377	-1.7410277	down	0.006318152
hsa-mir-491	-1.7368131	down	0.003009533
hsa-mir-639	-1.7293487	down	8.54E-04
hsa-mir-1181	-1.6956798	down	1.06E-04
hsa-mir-410	-1.6169846	down	0.035125908
hsa-mir-543	-1.6152669	down	0.002571987
hsa-mir-383	-1.6124716	down	0.009697244
hsa-mir-23b	-1.5909384	down	0.015085858
hsa-mir-146b	-1.5897484	down	0.01843226
hsa-mir-1291	-1.5848768	down	0.021788832
hsa-let-7i	-1.5837309	down	3.02E-04
hsa-mir-1234	-1.5805634	down	1.76E-17
hsa-mir-106b	-1.5497692	down	0.006169519
hsa-mir-24-1	-1.5300697	down	0.024964344
hsa-mir-138-1	-1.5150045	down	0.0032865
hsa-mir-374a	140.92953	up	0
hsa-mir-142	65.22522	up	1.82E-30
hsa-mir-19b-2	63.5723	up	0
hsa-mir-135b	60.82974	up	1.00E-23
hsa-mir-21	52.989597	up	0
hsa-mir-19a	45.7885	up	2.60E-27
hsa-mir-577	40.05445	up	6.27E-13
hsa-mir-424	30.184464	up	8.15E-33
hsa-mir-29b-1	30.046535	up	2.06E-38
hsa-mir-101-1	29.692446	up	0
hsa-mir-542	28.222824	up	0
hsa-mir-582	27.83786	up	8.70E-39
hsa-mir-708	24.78667	up	3.67E-36
hsa-mir-144	23.961945	up	7.08E-24
hsa-mir-141	22.687477	up	4.10E-37
hsa-mir-20a	22.144264	up	1.93E-30
hsa-mir-590	21.505735	up	1.94E-26
hsa-mir-153-2	20.456884	up	6.87E-17
hsa-mir-592	19.946692	up	8.58E-10
hsa-mir-335	19.592241	up	5.06E-34
hsa-mir-182	18.78644	up	3.18E-31
hsa-mir-452	17.161093	up	5.54E-30
hsa-mir-215	16.743689	up	3.05E-11
hsa-mir-552	16.048706	up	1.84E-09
hsa-mir-203	14.982542	up	8.11E-17
hsa-mir-379	14.027545	up	3.30E-36
hsa-mir-16-1	13.9177475	up	7.24E-24
hsa-mir-96	12.935062	up	3.83E-25
hsa-mir-889	12.8214655	up	6.96E-39
hsa-mir-98	12.206911	up	0
hsa-mir-136	11.756475	up	1.66E-17
hsa-mir-1-2	11.736584	up	4.75E-11

hsa-mir-374b	11.382514	up	1.24E-35
hsa-mir-628	11.372878	up	6.20E-11
hsa-mir-126	10.745708	up	2.87E-25
hsa-mir-217	10.295042	up	2.40E-14
hsa-mir-148a	9.867116	up	4.41E-20
hsa-mir-152	9.414362	up	7.31E-43
hsa-mir-301a	9.122609	up	9.49E-12
hsa-mir-450b	8.92358	up	6.22E-21
hsa-mir-429	8.92357	up	1.53E-16
hsa-mir-15a	8.417794	up	2.86E-28
hsa-mir-660	8.408914	up	7.37E-20
hsa-mir-32	7.9090858	up	7.04E-20
hsa-mir-454	7.865963	up	5.96E-27
hsa-mir-369	6.793081	up	2.11E-20
hsa-mir-17	6.714736	up	4.21E-16
hsa-mir-143	6.711155	up	3.83E-11
hsa-mir-30b	6.6690645	up	2.29E-16
hsa-mir-106a	6.2725577	up	3.07E-05
hsa-mir-411	6.2438	up	3.42E-15
hsa-mir-218-2	6.183688	up	6.93E-13
hsa-mir-7-1	6.1583357	up	3.69E-15
hsa-mir-10a	5.919755	up	1.32E-12
hsa-mir-199b	5.7847676	up	2.82E-10
hsa-mir-653	5.6058817	up	7.58E-07
hsa-mir-376c	5.4012823	up	2.63E-13
hsa-mir-337	5.3318877	up	1.32E-16
hsa-mir-192	5.2031627	up	1.62E-07
hsa-mir-598	5.127586	up	1.79E-09
hsa-mir-183	4.7046022	up	6.93E-10
hsa-mir-33a	4.453369	up	2.64E-05
hsa-mir-340	4.3543253	up	2.83E-16
hsa-mir-29c	4.2929397	up	3.25E-09
hsa-mir-421	4.2544603	up	4.89E-11
hsa-mir-95	4.1945605	up	3.55E-07
hsa-mir-495	4.146432	up	6.16E-16
hsa-mir-451	4.125477	up	4.40E-05
hsa-mir-223	4.0699043	up	9.68E-06
hsa-mir-24-2	3.9893742	up	5.30E-25
hsa-mir-31	3.9565177	up	0.005236639
hsa-mir-199a-2	3.7405434	up	4.16E-07
hsa-mir-188	3.7089033	up	2.01E-06
hsa-mir-27a	3.6482308	up	2.51E-10
hsa-mir-196a-1	3.6381824	up	1.30E-04
hsa-mir-450a-1	3.4919376	up	3.65E-09
hsa-mir-33b	3.4794478	up	7.89E-05
hsa-mir-338	3.4540892	up	1.69E-04
hsa-mir-26b	3.4343598	up	3.21E-11
hsa-mir-651	3.3484898	up	3.87E-08
hsa-mir-18a	3.2894936	up	5.87E-05
hsa-let-7f-1	3.2703521	up	7.14E-17
hsa-mir-194-1	3.245227	up	1.03E-07
hsa-mir-196b	3.2206805	up	0.003327127
hsa-mir-10b	3.2112288	up	3.02E-09
hsa-mir-190	3.1710253	up	3.17E-09
hsa-mir-30e	3.1080387	up	1.08E-15
hsa-mir-34a	3.0841117	up	5.19E-08
hsa-mir-224	2.9725883	up	1.64E-04
hsa-mir-301b	2.9368408	up	2.57E-05
hsa-mir-103-2	2.8811517	up	2.95E-05
hsa-mir-146a	2.8789024	up	1.24E-04
hsa-mir-151	2.7641008	up	1.22E-09
hsa-mir-496	2.6916192	up	7.60E-08
hsa-mir-26a-2	2.6861076	up	2.59E-11
hsa-mir-22	2.6556854	up	1.04E-13
hsa-mir-493	2.6089272	up	2.84E-08

hsa-mir-584	2.5641572	up	0.002599256
hsa-mir-508	2.5492427	up	0.025842048
hsa-mir-627	2.5214627	up	1.41E-05
hsa-mir-1977	2.500068	up	0.010464604
hsa-let-7g	2.4424646	up	8.04E-11
hsa-mir-376a-1	2.4373012	up	1.19E-06
hsa-mir-147b	2.410581	up	0.004115132
hsa-mir-1245	2.3560169	up	1.23E-06
hsa-mir-655	2.35559	up	1.61E-06
hsa-mir-181d	2.3057535	up	0.003218368
hsa-mir-1277	2.285632	up	2.55E-05
hsa-mir-376b	2.2767627	up	1.71E-05
hsa-mir-549	2.2236876	up	3.74E-05
hsa-mir-185	2.2018151	up	3.18E-07
hsa-mir-494	2.1872504	up	8.39E-06
hsa-mir-556	2.165163	up	9.81E-05
hsa-mir-576	2.1492126	up	8.88E-04
hsa-mir-412	2.1008253	up	0.012250098
hsa-mir-186	2.0487375	up	6.23E-05
hsa-mir-130a	2.014791	up	0.001836359
hsa-mir-499	1.8538002	up	0.011716682
hsa-mir-545	1.8008773	up	3.92E-04
hsa-mir-570	1.746147	up	8.08E-04
hsa-mir-643	1.7392558	up	1.64E-04
hsa-mir-548f-1	1.7317908	up	0.013023482
hsa-mir-758	1.7260778	up	0.008539054
hsa-mir-1308	1.7176796	up	0.013787789
hsa-mir-23a	1.7013396	up	4.70E-04
hsa-mir-107	1.6853368	up	4.09E-04
hsa-mir-380	1.6485493	up	4.16E-04
hsa-mir-216a	1.6447345	up	0.00721922
hsa-mir-200a	1.6173214	up	0.023149977
hsa-mir-559	1.6038537	up	0.002255896
hsa-mir-656	1.5602485	up	0.001615316
hsa-mir-25	1.5594497	up	0.009705679
hsa-mir-580	1.5435183	up	0.001934664
hsa-mir-382	1.5420406	up	0.013450535

Table S2. Differentially expressed miRNAs were analyzed in READ cancer versus normal tissue based on TCGA data. The threshold value for upregulated and downregulated genes was a fold change ≥1.5 and *p*-value ≤0.05.

Systematic Name	FC (abs)	Regulation	*p*
hsa-mir-490	-135.98697	down	6.59E-13
hsa-mir-328	-134.68758	down	4.40E-37
hsa-mir-139	-80.28687	down	1.06E-30
hsa-mir-504	-76.078545	down	0
hsa-mir-486	-70.63095	down	1.51E-14
hsa-mir-133a-1	-64.6055	down	3.18E-13
hsa-mir-149	-59.165817	down	5.27E-20
hsa-mir-129-1	-56.821983	down	2.61E-31
hsa-mir-1224	-55.13707	down	5.54E-30
hsa-mir-197	-47.221104	down	3.44E-27
hsa-mir-574	-46.144466	down	4.35E-23
hsa-mir-766	-42.3317	down	3.27E-30
hsa-mir-145	-36.196594	down	1.82E-15
hsa-mir-193a	-30.22148	down	3.59E-18
hsa-mir-125a	-29.290022	down	8.03E-23
hsa-mir-1306	-27.28638	down	2.49E-20
hsa-mir-1180	-26.601833	down	4.06E-12
hsa-let-7d	-24.964872	down	1.05E-34
hsa-mir-150	-23.079159	down	1.77E-12
hsa-mir-1296	-21.62183	down	3.60E-16
hsa-mir-92b	-19.150013	down	2.37E-12

hsa-mir-1249	-18.639511	down	9.40E-22
hsa-mir-99b	-18.635223	down	1.98E-22
hsa-mir-125b-1	-15.973903	down	2.26E-08
hsa-mir-1976	-15.401709	down	5.95E-17
hsa-mir-485	-14.917463	down	1.50E-14
hsa-mir-605	-14.679367	down	1.30E-23
hsa-mir-193b	-14.586462	down	6.73E-11
hsa-mir-1226	-12.453842	down	6.76E-17
hsa-mir-326	-11.861671	down	1.06E-11
hsa-let-7b	-11.739235	down	1.37E-18
hsa-mir-433	-11.2762165	down	5.68E-17
hsa-mir-133b	-10.657723	down	1.09E-06
hsa-mir-375	-10.57266	down	6.03E-05
hsa-let-7c	-10.388263	down	2.04E-05
hsa-mir-760	-9.92051	down	1.02E-12
hsa-mir-296	-9.388603	down	1.38E-04
hsa-mir-370	-9.000886	down	2.03E-13
hsa-mir-378	-8.737416	down	3.01E-09
hsa-mir-432	-8.663443	down	2.25E-09
hsa-mir-2110	-8.566132	down	7.78E-11
hsa-mir-205	-8.013963	down	0.001158812
hsa-mir-363	-7.9215703	down	1.39E-06
hsa-mir-181a-1	-7.5249705	down	2.84E-09
hsa-mir-589	-7.2122483	down	1.42E-11
hsa-mir-423	-7.169264	down	1.15E-11
hsa-mir-874	-6.751404	down	1.76E-05
hsa-mir-1468	-6.6051564	down	5.74E-09
hsa-mir-324	-5.883902	down	4.73E-10
hsa-mir-431	-5.862475	down	3.98E-08
hsa-mir-885	-5.8277392	down	1.74E-11
hsa-mir-937	-5.8269687	down	1.45E-05
hsa-mir-744	-5.642323	down	9.82E-07
hsa-mir-187	-5.6009455	down	2.28E-05
hsa-mir-92a-2	-5.2479057	down	3.31E-06
hsa-mir-383	-5.2167277	down	1.09E-09
hsa-mir-346	-5.172708	down	1.88E-31
hsa-mir-331	-5.1329236	down	3.10E-04
hsa-mir-671	-4.994799	down	4.12E-10
hsa-mir-1229	-4.9548635	down	3.95E-10
hsa-mir-484	-4.899485	down	5.18E-07
hsa-mir-877	-4.6133723	down	6.59E-05
hsa-mir-365-1	-4.584179	down	6.02E-06
hsa-mir-323	-4.5467825	down	1.15E-05
hsa-mir-1538	-4.4786053	down	1.94E-27
hsa-mir-891a	-4.400809	down	2.98E-05
hsa-mir-505	-4.2703285	down	4.56E-06
hsa-mir-1228	-4.2165804	down	2.68E-08
hsa-mir-320c-1	-4.0962973	down	2.64E-11
hsa-mir-20b	-4.0940976	down	0.001262076
hsa-mir-339	-4.0692787	down	2.16E-05
hsa-mir-1307	-3.9854705	down	9.67E-05
hsa-mir-140	-3.7967339	down	2.10E-06
hsa-mir-1271	-3.618777	down	1.26E-04
hsa-mir-887	-3.5285904	down	2.61E-06
hsa-mir-342	-3.491284	down	3.90E-04
hsa-mir-15b	-3.4353487	down	4.73E-05
hsa-mir-214	-3.3951535	down	9.14E-04
hsa-mir-487a	-3.3621833	down	7.58E-06
hsa-mir-132	-3.224012	down	2.16E-05
hsa-mir-28	-3.1282322	down	7.89E-09
hsa-mir-127	-2.8960972	down	1.49E-04
hsa-mir-658	-2.8960013	down	5.57E-10
hsa-mir-663b	-2.8168721	down	0.003313308
hsa-mir-135a-1	-2.7820208	down	3.24E-06
hsa-mir-1292	-2.759025	down	3.55E-05

hsa-mir-455	-2.751464	down	0.016525857
hsa-mir-501	-2.7253783	down	0.014389883
hsa-mir-1301	-2.684648	down	0.001770045
hsa-mir-1234	-2.5178773	down	3.95E-30
hsa-mir-939	-2.4398868	down	8.76E-05
hsa-mir-1287	-2.4396005	down	0.002906155
hsa-mir-632	-2.3584	down	6.96E-05
hsa-mir-1237	-2.326263	down	1.88E-22
hsa-mir-18b	-2.3011756	down	0.001028637
hsa-mir-361	-2.292101	down	0.002301976
hsa-mir-202	-2.2430816	down	2.53E-05
hsa-mir-1227	-2.2043328	down	9.34E-10
hsa-mir-30d	-2.183335	down	0.003102631
hsa-mir-497	-2.1412995	down	0.0128487
hsa-mir-491	-2.1318836	down	0.006817964
hsa-mir-377	-1.980699	down	0.017571513
hsa-mir-1181	-1.9599565	down	4.43E-04
hsa-mir-487b	-1.9505255	down	0.008613803
hsa-mir-218-1	-1.8999251	down	2.46E-04
hsa-mir-106b	-1.8719217	down	0.011739618
hsa-mir-138-1	-1.727772	down	0.014360772
hsa-mir-770	-1.7002965	down	7.84E-07
hsa-mir-329-2	-1.6802279	down	0.006776016
hsa-mir-1910	-1.5881958	down	1.66E-07
hsa-mir-1908	-1.5731761	down	9.39E-09
hsa-mir-1914	-1.5566725	down	0.001414808
hsa-mir-552	125.86824	up	2.73E-15
hsa-mir-135b	92.44072	up	3.89E-12
hsa-mir-424	87.797104	up	4.46E-19
hsa-mir-215	74.0141	up	3.09E-08
hsa-mir-142	73.418945	up	3.29E-17
hsa-mir-19b-2	68.83364	up	3.83E-20
hsa-mir-374a	67.5058	up	2.60E-27
hsa-mir-577	63.387184	up	2.07E-08
hsa-mir-19a	60.3911	up	8.56E-13
hsa-mir-21	54.890163	up	0
hsa-mir-20a	46.65114	up	9.34E-22
hsa-mir-144	42.5793	up	7.57E-12
hsa-mir-335	41.66285	up	6.43E-22
hsa-mir-452	38.607204	up	1.10E-18
hsa-mir-141	36.88312	up	1.42E-23
hsa-mir-429	35.916325	up	2.97E-16
hsa-mir-16-1	33.6399	up	9.60E-19
hsa-mir-203	33.5487	up	7.20E-13
hsa-mir-192	30.339136	up	2.72E-16
hsa-mir-126	29.031384	up	1.72E-21
hsa-mir-542	28.02535	up	1.38E-19
hsa-mir-29b-1	27.505907	up	1.02E-17
hsa-mir-592	27.477612	up	8.84E-07
hsa-mir-590	26.95451	up	5.12E-19
hsa-mir-148a	26.470291	up	5.77E-16
hsa-mir-182	24.657486	up	1.95E-14
hsa-mir-15a	24.0329	up	9.63E-23
hsa-mir-98	24.021187	up	8.69E-22
hsa-mir-101-1	23.364975	up	1.00E-21
hsa-mir-379	21.32783	up	2.48E-21
hsa-mir-374b	20.26702	up	3.46E-21
hsa-mir-194-1	17.759962	up	9.22E-16
hsa-mir-1-2	17.396029	up	3.00E-06
hsa-mir-660	16.94982	up	1.77E-17
hsa-mir-889	16.367073	up	6.09E-20
hsa-mir-628	15.807961	up	2.10E-07
hsa-mir-224	15.315301	up	5.17E-11
hsa-mir-17	14.323981	up	2.19E-15
hsa-mir-153-2	14.219922	up	1.28E-07

hsa-mir-196a-1	12.448104	up	7.73E-07
hsa-mir-10a	12.150859	up	6.33E-10
hsa-mir-217	12.046613	up	1.14E-06
hsa-mir-196b	11.582685	up	9.77E-05
hsa-mir-301a	11.0168915	up	6.16E-07
hsa-mir-95	10.984338	up	3.75E-08
hsa-mir-96	10.834814	up	1.67E-10
hsa-mir-454	10.746106	up	9.01E-18
hsa-mir-450b	10.014402	up	2.35E-09
hsa-mir-582	9.51795	up	1.33E-10
hsa-mir-183	9.238996	up	5.80E-08
hsa-mir-369	9.099438	up	1.45E-10
hsa-mir-411	9.001889	up	3.77E-09
hsa-mir-223	8.863569	up	5.08E-06
hsa-mir-508	8.845228	up	0.001173209
hsa-mir-708	8.785184	up	3.78E-08
hsa-let-7f-1	8.779806	up	1.09E-09
hsa-mir-152	8.365665	up	4.48E-15
hsa-mir-32	7.9362955	up	8.08E-08
hsa-mir-7-1	7.756975	up	7.40E-08
hsa-mir-188	7.717349	up	8.94E-07
hsa-mir-451	7.3666644	up	3.86E-04
hsa-mir-10b	7.211374	up	4.30E-09
hsa-mir-31	7.122003	up	0.00565925
hsa-mir-34a	6.9761076	up	1.53E-08
hsa-mir-33a	6.971081	up	6.37E-05
hsa-mir-146a	6.138518	up	5.39E-05
hsa-mir-30b	6.1113443	up	3.06E-07
hsa-mir-151	6.0227947	up	3.14E-13
hsa-mir-199b	5.7899895	up	3.06E-05
hsa-mir-653	5.566485	up	1.03E-04
hsa-mir-103-2	5.565713	up	1.90E-05
hsa-mir-218-2	5.5261154	up	2.65E-05
hsa-mir-18a	5.462307	up	4.73E-04
hsa-mir-136	5.3182235	up	1.84E-05
hsa-mir-376c	5.22864	up	2.40E-06
hsa-mir-493	5.147699	up	1.37E-08
hsa-let-7g	4.9600925	up	1.14E-09
hsa-mir-24-2	4.919142	up	3.26E-12
hsa-mir-26b	4.8252816	up	5.89E-08
hsa-mir-29c	4.5677586	up	1.63E-05
hsa-mir-7-2	4.5404496	up	3.12E-04
hsa-mir-421	4.5337253	up	7.76E-05
hsa-mir-495	4.509793	up	1.13E-07
hsa-mir-27a	4.4260273	up	3.79E-06
hsa-mir-22	4.28322	up	1.65E-10
hsa-mir-186	4.23124	up	2.52E-09
hsa-mir-143	4.219866	up	2.92E-04
hsa-mir-200b	4.196607	up	3.01E-06
hsa-mir-26a-2	4.137692	up	3.06E-09
hsa-mir-33b	4.0763574	up	0.005779905
hsa-mir-425	4.065508	up	7.59E-04
hsa-mir-503	4.0478563	up	0.006464281
hsa-mir-181d	4.011771	up	9.89E-04
hsa-mir-651	3.996633	up	9.98E-06
hsa-mir-185	3.954062	up	3.96E-09
hsa-mir-190	3.8318734	up	1.48E-05
hsa-mir-450a-2	3.814068	up	4.54E-05
hsa-mir-155	3.749655	up	2.49E-04
hsa-mir-199a-2	3.7423735	up	5.95E-04
hsa-mir-200a	3.6802692	up	2.47E-04
hsa-mir-130a	3.6583018	up	2.48E-05
hsa-mir-107	3.6367493	up	4.38E-09
hsa-mir-338	3.5948071	up	0.010991716
hsa-mir-7-3	3.572123	up	0.002919351

hsa-mir-340	3.545447	up	3.07E-06
hsa-mir-301b	3.5284646	up	0.001478936
hsa-mir-627	3.3326344	up	8.22E-05
hsa-mir-30e	3.2756462	up	3.80E-07
hsa-mir-381	2.8574576	up	3.26E-04
hsa-mir-1277	2.7795572	up	3.51E-04
hsa-mir-376b	2.7681174	up	4.91E-04
hsa-mir-655	2.7593389	up	3.42E-04
hsa-mir-337	2.7227244	up	2.09E-04
hsa-mir-576	2.7083774	up	0.002856645
hsa-mir-376a-1	2.650357	up	3.30E-04
hsa-mir-496	2.5329292	up	0.003536142
hsa-mir-1245	2.3480031	up	0.002196955
hsa-mir-556	2.3231072	up	0.006246795
hsa-mir-549	2.3110714	up	0.011851881
hsa-mir-29a	2.275791	up	0.002045545
hsa-mir-758	2.2667346	up	0.005095392
hsa-mir-25	2.1846185	up	0.003145347
hsa-mir-23a	1.9521618	up	0.011320274
hsa-mir-570	1.901412	up	0.012359721
hsa-mir-624	1.8810266	up	0.00555362
hsa-mir-643	1.7392855	up	0.012790618
hsa-mir-380	1.7313354	up	0.007913949

Table S3. Differentially expressed miRNAs were analyzed in CRC cancer versus normal tissue based on TCGA data. The threshold value for upregulated and downregulated genes was a fold change ≥1.5 and *p*-value ≤0.05.

Systematic Name	FC (abs)	Regulation	*p*
hsa-miR-129-1	-4.858034	down	0
hsa-miR-504	-4.7633567	down	0
hsa-miR-1224	-4.7087784	down	0
hsa-miR-490	-3.7090964	down	7.81E-15
hsa-miR-642	-3.4915624	down	9.04E-18
hsa-miR-1226	-3.4715407	down	8.96E-41
hsa-miR-1249	-3.2612455	down	2.54E-36
hsa-miR-433	-3.2496326	down	8.00E-39
hsa-miR-605	-3.179483	down	0
hsa-miR-2110	-2.8325794	down	3.50E-21
hsa-miR-485	-2.8254273	down	5.03E-20
hsa-miR-766	-2.7934394	down	3.71E-30
hsa-miR-760	-2.7578673	down	1.37E-25
hsa-miR-767	-2.7436526	down	2.46E-08
hsa-miR-205	-2.739815	down	6.83E-10
hsa-miR-1296	-2.6734877	down	1.50E-17
hsa-miR-296	-2.64368	down	1.74E-09
hsa-miR-483	-2.6117496	down	1.85E-05
hsa-miR-1228	-2.6051078	down	4.97E-37
hsa-miR-1306	-2.6021512	down	4.30E-24
hsa-miR-326	-2.5510483	down	4.50E-14
hsa-miR-1976	-2.5414922	down	4.42E-26
hsa-miR-328	-2.5343375	down	6.24E-41
hsa-miR-149	-2.5233283	down	2.10E-19
hsa-miR-937	-2.5119572	down	2.37E-12
hsa-miR-1180	-2.4826884	down	2.38E-15
hsa-miR-133b	-2.419263	down	3.17E-08
hsa-miR-885	-2.372808	down	0
hsa-miR-487a	-2.3506322	down	1.34E-21
hsa-miR-1468	-2.291185	down	1.84E-13
hsa-miR-125b-2	-2.2844684	down	6.33E-09
hsa-miR-139	-2.251541	down	0
hsa-miR-887	-2.2088375	down	8.52E-15
hsa-miR-204	-2.207423	down	1.59E-06
hsa-miR-187	-2.1849632	down	1.79E-11

hsa-miR-1229	-2.1762161	down	6.20E-36
hsa-miR-486	-2.138717	down	1.53E-25
hsa-miR-363	-2.1325169	down	1.33E-07
hsa-miR-432	-2.129805	down	2.46E-12
hsa-miR-133a-2	-2.076548	down	1.10E-13
hsa-miR-1538	-2.073734	down	0
hsa-miR-877	-2.0561497	down	6.97E-09
hsa-miR-323	-2.0124588	down	2.50E-09
hsa-miR-891a	-2.009999	down	9.87E-11
hsa-miR-1271	-1.9774262	down	2.92E-08
hsa-miR-671	-1.9576324	down	3.41E-19
hsa-miR-1275	-1.8874406	down	4.15E-05
hsa-miR-1270	-1.884741	down	4.46E-31
hsa-miR-320c-1	-1.873387	down	0
hsa-miR-935	-1.8306041	down	4.12E-04
hsa-miR-370	-1.8170676	down	2.19E-13
hsa-miR-1254	-1.7977576	down	1.29E-20
hsa-miR-574	-1.7972102	down	2.25E-32
hsa-miR-615	-1.7908909	down	4.36E-07
hsa-miR-874	-1.7782515	down	1.68E-10
hsa-miR-218-1	-1.7708175	down	0
hsa-miR-939	-1.7590865	down	4.61E-10
hsa-miR-105-2	-1.7466513	down	0.002273916
hsa-miR-193b	-1.719887	down	6.08E-18
hsa-miR-346	-1.7060112	down	0
hsa-miR-1292	-1.6991967	down	1.26E-19
hsa-miR-383	-1.6690658	down	4.24E-12
hsa-miR-197	-1.6630012	down	0
hsa-miR-431	-1.6581236	down	5.92E-11
hsa-miR-92b	-1.6517613	down	1.37E-22
hsa-miR-1266	-1.6463609	down	1.64E-05
hsa-miR-1301	-1.6365737	down	5.30E-07
hsa-miR-1248	-1.6362013	down	1.76E-04
hsa-miR-663b	-1.6209098	down	3.81E-05
hsa-miR-193a	-1.5834945	down	8.72E-28
hsa-miR-1274b	-1.5763532	down	0.003775847
hsa-miR-219-1	-1.5725294	down	2.62E-05
hsa-miR-632	-1.5692668	down	7.78E-24
hsa-miR-324	-1.556021	down	5.21E-21
hsa-miR-125a	-1.5363654	down	0
hsa-miR-20b	-1.5359448	down	0.004503305
hsa-miR-150	-1.5326179	down	5.94E-20
hsa-miR-365-1	-1.5111432	down	2.19E-07
hsa-miR-744	-1.5052631	down	8.71E-14
hsa-miR-135b	5.9248714	up	0
hsa-miR-19a	5.448208	up	0
hsa-miR-577	5.0403543	up	1.31E-35
hsa-miR-19b-2	4.7023287	up	0
hsa-miR-590	4.478734	up	0
hsa-miR-144	4.373971	up	0
hsa-miR-424	4.232565	up	0
hsa-miR-592	3.966677	up	4.23E-17
hsa-miR-153-2	3.82902	up	7.93E-40
hsa-miR-708	3.670745	up	0
hsa-miR-889	3.6557608	up	0
hsa-miR-101-2	3.557317	up	0
hsa-miR-628	3.5299652	up	6.44E-27
hsa-miR-374a	3.5115545	up	0
hsa-miR-96	3.505476	up	0
hsa-miR-1-2	3.219226	up	1.60E-27
hsa-miR-542	3.1603498	up	0
hsa-miR-301a	3.1394703	up	3.34E-22
hsa-miR-217	3.1058514	up	7.24E-35
hsa-miR-454	3.0999076	up	2.80E-45
hsa-miR-98	3.0937862	up	0

hsa-miR-552	3.090351	up	2.25E-24
hsa-miR-450b	3.073356	up	3.66E-31
hsa-miR-452	3.0206714	up	0
hsa-miR-369	2.8473043	up	2.78E-42
hsa-miR-136	2.7963743	up	3.15E-39
hsa-miR-411	2.7473662	up	1.24E-22
hsa-miR-374b	2.6867683	up	0
hsa-miR-660	2.679474	up	0
hsa-miR-335	2.526943	up	0
hsa-miR-218-2	2.509361	up	1.89E-23
hsa-miR-495	2.4881825	up	1.09E-27
hsa-miR-376c	2.452837	up	7.97E-16
hsa-miR-32	2.4112337	up	2.00E-44
hsa-miR-95	2.3634226	up	1.30E-16
hsa-miR-29b-1	2.3240824	up	0
hsa-miR-653	2.2456872	up	9.96E-08
hsa-miR-142	2.2234454	up	0
hsa-miR-188	2.2178683	up	3.40E-11
hsa-miR-421	2.177643	up	7.40E-12
hsa-miR-20a	2.1504717	up	0
hsa-miR-582	2.1463692	up	0
hsa-miR-215	2.1386993	up	6.95E-31
hsa-miR-15a	2.1372988	up	0
hsa-miR-190	2.114514	up	3.01E-15
hsa-miR-31	2.084972	up	1.35E-04
hsa-miR-598	2.0749962	up	3.47E-12
hsa-let-7f-1	2.0264852	up	2.76E-36
hsa-miR-106a	2.0253804	up	3.65E-09
hsa-miR-33b	2.0148382	up	2.55E-06
hsa-miR-340	1.9595792	up	3.84E-29
hsa-miR-7-1	1.9589001	up	3.74E-34
hsa-miR-337	1.9362608	up	2.85E-31
hsa-miR-1977	1.8962864	up	6.47E-07
hsa-miR-493	1.8850685	up	3.66E-23
hsa-miR-508	1.8649338	up	1.21E-04
hsa-miR-7-2	1.8547143	up	2.84E-06
hsa-miR-103-2	1.8396531	up	1.13E-12
hsa-miR-16-1	1.8114393	up	0
hsa-miR-429	1.7983998	up	4.12E-42
hsa-miR-450a-2	1.7933439	up	4.47E-07
hsa-miR-651	1.7899704	up	9.32E-07
hsa-miR-26a-1	1.7781942	up	0.001317235
hsa-miR-7-3	1.7778119	up	1.64E-05
hsa-miR-141	1.7770268	up	0
hsa-miR-33a	1.7433052	up	1.67E-10
hsa-miR-379	1.7290301	up	0
hsa-miR-152	1.728595	up	0
hsa-miR-18a	1.661251	up	4.63E-09
hsa-miR-301b	1.6407888	up	2.43E-04
hsa-miR-496	1.6023065	up	1.22E-05
hsa-miR-224	1.582061	up	2.48E-12
hsa-miR-627	1.5594852	up	1.75E-04
hsa-miR-576	1.5593785	up	2.31E-07
hsa-miR-126	1.5329307	up	0
hsa-miR-181d	1.5215815	up	1.41E-05
hsa-miR-147b	1.5213158	up	0.004936536

Table S4. Differentially expressed miRNAs were analyzed in plasma colon cancer patients versus healthy controls. The threshold value for upregulated and downregulated genes was a fold change ≥1.5 and *p*-value ≤0.05.

Systematic_Name	FC (abs)	Regulation	*p*
hsa-miR-195-5p	-19.084541	down	6.69E-15
hsa-miR-96-5p	-17.871162	down	7.25E-17

hsa-miR-363-3p	-17.0228	down	4.95E-07
hsa-miR-4741	-15.618946	down	9.97E-09
hsa-miR-642b-3p	-14.030791	down	1.21E-06
hsa-miR-374a-5p	-12.953635	down	6.50E-08
hsa-miR-192-5p	-12.524868	down	1.77E-09
hsa-miR-151a-5p	-12.284961	down	2.13E-08
hsa-miR-660-5p	-12.08418	down	6.03E-08
hsa-miR-134-5p	-11.779961	down	1.47E-07
hsa-miR-301a-3p	-11.585538	down	5.73E-10
hsa-miR-6068	-11.114471	down	1.24E-05
hsa-miR-18a-5p	-11.00917	down	6.07E-09
hsa-miR-30b-5p	-10.96	down	1.44E-06
hsa-miR-126-5p	-10.817145	down	1.27E-09
hsa-miR-4728-5p	-10.660697	down	6.34E-17
hsa-miR-151b	-10.577785	down	3.83E-09
hsa-miR-590-5p	-10.264501	down	1.38E-07
hsa-miR-6728-5p	-9.927017	down	7.78E-08
hsa-miR-148a-3p	-9.923865	down	1.81E-06
hsa-miR-5739	-9.6984415	down	1.32E-05
hsa-miR-425-5p	-9.599833	down	4.94E-05
hsa-miR-3135b	-9.34744	down	2.74E-07
hsa-miR-142-5p	-9.205788	down	6.80E-05
hsa-miR-27b-3p	-9.011637	down	8.58E-06
hsa-miR-215-5p	-8.917941	down	2.83E-10
hsa-miR-1227-5p	-8.911604	down	9.05E-07
hsa-miR-371b-5p	-8.890483	down	2.09E-04
hsa-miR-186-5p	-8.877	down	9.09E-08
hsa-miR-148b-3p	-8.662281	down	8.12E-09
hsa-miR-575	-8.462271	down	1.97E-07
hsa-miR-4530	-8.448288	down	0.002184228
hsa-miR-642a-3p	-8.283251	down	0.005282057
hsa-miR-324-3p	-8.146081	down	2.03E-08
hsa-miR-4788	-8.133066	down	6.17E-10
hsa-miR-4534	-8.124963	down	5.27E-04
hsa-miR-7-5p	-8.020097	down	2.11E-11
hsa-miR-5787	-8.016673	down	0.00282615
hsa-miR-6791-5p	-7.9281197	down	7.65E-04
hsa-miR-4634	-7.9260564	down	4.83E-05
hsa-miR-3138	-7.6574693	down	8.34E-09
hsa-miR-331-3p	-7.551329	down	3.39E-09
hsa-miR-4442	-7.527234	down	7.49E-06
hsa-miR-6085	-7.512024	down	1.05E-05
hsa-miR-4443	-7.369146	down	6.40E-04
hsa-miR-4739	-7.366848	down	5.27E-06
hsa-miR-30c-5p	-7.317877	down	2.99E-08
hsa-miR-26b-5p	-7.309291	down	2.83E-04
hsa-miR-6789-5p	-7.2860436	down	1.23E-08
hsa-miR-183-5p	-7.085235	down	1.05E-13
hsa-miR-374b-5p	-6.9002233	down	3.86E-09
hsa-miR-18b-5p	-6.844817	down	9.97E-09
hsa-miR-7108-5p	-6.841108	down	1.42E-04
hsa-miR-8072	-6.722614	down	0.001054136
hsa-miR-98-5p	-6.7059298	down	2.56E-16
hsa-miR-5585-3p	-6.674031	down	7.27E-09
hsa-miR-572	-6.480188	down	1.77E-07
hsa-miR-128-3p	-6.4210453	down	2.11E-12
hsa-miR-3195	-6.395294	down	7.95E-09
hsa-miR-23b-3p	-6.2139225	down	1.23E-05
hsa-miR-630	-6.2048616	down	0.021493422
hsa-miR-6786-5p	-6.180855	down	1.71E-05
hsa-miR-5703	-6.180663	down	0.020989262
hsa-miR-6869-5p	-6.153493	down	0.013190362
hsa-miR-6875-5p	-6.092739	down	4.08E-08
hsa-miR-939-5p	-6.036088	down	2.99E-07
hsa-miR-3679-5p	-6.0327153	down	0.001908814

hsa-miR-6752-5p	-5.923568	down	3.93E-04
hsa-miR-718	-5.911115	down	2.64E-04
hsa-miR-188-5p	-5.8806696	down	3.42E-13
hsa-miR-1471	-5.8774614	down	2.46E-07
hsa-miR-6850-5p	-5.8709145	down	0.011368866
hsa-miR-6780b-5p	-5.8104053	down	4.86E-08
hsa-miR-4459	-5.7814665	down	0.017793614
hsa-miR-454-3p	-5.7681375	down	2.44E-14
hsa-miR-4651	-5.7668676	down	1.25E-13
hsa-miR-5006-5p	-5.750572	down	1.56E-06
hsa-miR-199a-5p	-5.7422247	down	4.67E-04
hsa-miR-144-5p	-5.700877	down	2.15E-09
hsa-miR-3940-5p	-5.677904	down	3.45E-05
hsa-miR-140-5p	-5.6675124	down	4.98E-07
hsa-miR-6749-5p	-5.618565	down	0.009301984
hsa-miR-1183	-5.497882	down	8.80E-08
hsa-miR-8063	-5.4752316	down	1.17E-06
hsa-miR-7641	-5.4738154	down	2.48E-04
hsa-miR-146a-5p	-5.334993	down	8.21E-04
hsa-miR-3188	-5.332079	down	1.62E-07
hsa-miR-638	-5.2982693	down	0.014095136
hsa-miR-130b-3p	-5.2672663	down	1.66E-04
hsa-miR-342-3p	-5.1831193	down	0.001545311
hsa-miR-6763-5p	-5.144915	down	1.65E-05
hsa-miR-1202	-5.112082	down	0.006015135
hsa-miR-6893-5p	-5.0950265	down	1.37E-10
hsa-miR-150-3p	-5.0198402	down	4.19E-08
hsa-miR-4484	-5.0191875	down	1.16E-07
hsa-miR-340-5p	-4.9916167	down	4.23E-09
hsa-miR-1207-5p	-4.966287	down	0.012024546
hsa-miR-6829-5p	-4.956853	down	1.50E-10
hsa-miR-3196	-4.953924	down	1.57E-05
hsa-miR-548q	-4.855887	down	6.94E-11
hsa-miR-4463	-4.8432555	down	7.50E-04
hsa-miR-424-5p	-4.806207	down	1.28E-05
hsa-miR-3648	-4.7944536	down	1.03E-10
hsa-miR-1915-3p	-4.7243605	down	0.016082237
hsa-miR-125a-5p	-4.711557	down	2.55E-08
hsa-miR-3652	-4.687598	down	3.71E-06
hsa-miR-4271	-4.6779494	down	1.36E-07
hsa-miR-532-5p	-4.670084	down	5.11E-11
hsa-miR-4270	-4.488763	down	0.012020052
hsa-miR-671-5p	-4.4687114	down	1.77E-07
hsa-miR-6127	-4.415358	down	0.002855521
hsa-miR-10b-5p	-4.410356	down	4.98E-09
hsa-miR-6724-5p	-4.406721	down	0.009369723
hsa-miR-6800-5p	-4.393602	down	0.02623327
hsa-let-7d-3p	-4.388507	down	3.35E-04
hsa-miR-5195-3p	-4.352574	down	4.23E-06
hsa-miR-4327	-4.319587	down	1.51E-05
hsa-miR-484	-4.312819	down	3.51E-04
hsa-miR-210-3p	-4.240125	down	1.94E-06
hsa-miR-6812-5p	-4.2040324	down	0.001743548
hsa-miR-4721	-4.1983414	down	4.71E-09
hsa-miR-1225-5p	-4.171139	down	0.021552803
hsa-miR-3937	-4.1268516	down	1.15E-12
hsa-miR-3194-5p	-4.124269	down	1.31E-10
hsa-miR-6831-5p	-4.1073184	down	0.009396043
hsa-miR-3663-3p	-4.0961175	down	0.011477044
hsa-miR-345-3p	-4.077024	down	1.65E-09
hsa-miR-3667-5p	-4.059849	down	3.52E-05
hsa-miR-6858-5p	-4.037533	down	4.46E-09
hsa-miR-181a-5p	-4.024414	down	2.87E-04
hsa-miR-4745-5p	-4.015967	down	5.94E-10
hsa-miR-1914-3p	-3.9975688	down	8.70E-18

hsa-miR-4499	-3.9906962	down	2.26E-04
hsa-miR-6879-5p	-3.9535654	down	0.003344605
hsa-miR-3141	-3.8921163	down	0.006257298
hsa-miR-486-3p	-3.861394	down	7.13E-12
hsa-miR-335-5p	-3.8538697	down	3.49E-12
hsa-miR-6727-5p	-3.8398433	down	0.023623873
hsa-miR-6794-5p	-3.8389106	down	0.00273704
hsa-miR-2276-3p	-3.8282197	down	9.93E-09
hsa-miR-376a-3p	-3.7756028	down	7.74E-07
hsa-miR-4466	-3.7644992	down	0.029209772
hsa-miR-4787-5p	-3.756085	down	0.012493689
hsa-miR-584-5p	-3.7494311	down	5.30E-07
hsa-miR-937-5p	-3.7420526	down	2.52E-04
hsa-miR-6126	-3.707313	down	3.58E-08
hsa-miR-3610	-3.7047298	down	1.97E-13
hsa-miR-1268b	-3.6549137	down	2.13E-04
hsa-miR-7847-3p	-3.6157413	down	0.0357677
hsa-miR-6740-5p	-3.6133046	down	0.025256215
hsa-miR-16-2-3p	-3.5953636	down	0.001419533
hsa-miR-19a-3p	-3.5838585	down	0.004304006
hsa-miR-197-5p	-3.5818837	down	0.028940076
hsa-miR-8064	-3.5633283	down	4.90E-06
hsa-miR-6124	-3.514801	down	0.033647396
hsa-miR-29b-3p	-3.478355	down	0.007268644
hsa-miR-146b-5p	-3.4364433	down	1.04E-08
hsa-miR-135a-3p	-3.4224482	down	1.48E-04
hsa-miR-17-3p	-3.4116976	down	3.45E-04
hsa-miR-1249-5p	-3.373955	down	8.02E-07
hsa-miR-30e-5p	-3.3489783	down	0.01385989
hsa-miR-6086	-3.3278747	down	1.13E-05
hsa-miR-762	-3.3056538	down	0.014431517
hsa-miR-4778-5p	-3.2959955	down	0.00267932
hsa-miR-27a-3p	-3.2275836	down	0.030195076
hsa-miR-7845-5p	-3.21904	down	1.24E-07
hsa-miR-15b-5p	-3.204382	down	0.001744283
hsa-let-7g-5p	-3.1887221	down	0.001677265
hsa-miR-194-5p	-3.186671	down	2.10E-07
hsa-miR-378i	-3.1792	down	1.36E-04
hsa-miR-221-3p	-3.1278567	down	0.024818126
hsa-let-7c-5p	-3.1041744	down	0.01855364
hsa-miR-2861	-3.0810797	down	0.03618509
hsa-miR-6756-5p	-3.0367317	down	9.60E-04
hsa-miR-4707-3p	-3.0173829	down	5.37E-09
hsa-miR-20b-5p	-2.9449818	down	0.002139462
hsa-let-7f-5p	-2.8318105	down	0.02224775
hsa-miR-10a-5p	-2.8141494	down	1.80E-08
hsa-miR-4800-5p	-2.780537	down	0.019083614
hsa-miR-7106-5p	-2.7090771	down	0.01492428
hsa-miR-338-3p	-2.6697903	down	6.17E-04
hsa-miR-652-5p	-2.6586297	down	8.93E-11
hsa-miR-328-3p	-2.6507037	down	0.003074694
hsa-miR-198	-2.6403155	down	0.006110552
hsa-miR-125b-5p	-2.6154964	down	1.75E-04
hsa-miR-498	-2.556956	down	8.03E-06
hsa-miR-6073	-2.5482113	down	7.30E-06
hsa-miR-361-3p	-2.5060644	down	5.56E-11
hsa-miR-106b-5p	-2.4979243	down	0.016352372
hsa-let-7a-5p	-2.4907188	down	0.03822139
hsa-miR-6730-5p	-2.4802718	down	1.60E-11
hsa-miR-378a-3p	-2.4710562	down	2.70E-04
hsa-miR-30a-5p	-2.3956597	down	0.03168892
hsa-miR-6075	-2.3760273	down	1.43E-07
hsa-miR-4429	-2.3728902	down	3.18E-04
hsa-miR-365a-3p	-2.364505	down	3.29E-06
hsa-miR-3158-5p	-2.3610487	down	2.51E-12

hsa-miR-6820-5p	-2.3358476	down	3.76E-04
hsa-miR-34a-5p	-2.3222978	down	4.62E-04
hsa-miR-557	-2.2975543	down	5.81E-04
hsa-miR-497-5p	-2.2728772	down	7.18E-07
hsa-miR-623	-2.2431304	down	7.10E-04
hsa-miR-6790-5p	-2.2431083	down	3.62E-05
hsa-let-7i-5p	-2.2187643	down	1.23E-04
hsa-miR-222-3p	-2.1863503	down	0.036298715
hsa-miR-100-5p	-2.1320403	down	2.01E-06
hsa-miR-4481	-2.0843182	down	3.13E-10
hsa-miR-15a-5p	-2.0705006	down	2.32E-04
hsa-miR-25-3p	-2.0666978	down	0.003317509
hsa-miR-20a-5p	-2.0474055	down	0.001212804
hsa-miR-19b-3p	-2.0025215	down	0.001944795
hsa-miR-139-3p	-1.8184377	down	4.51E-09
hsa-miR-629-5p	-1.7804717	down	9.00E-05
hsa-miR-3960	-1.5608507	down	0.031699736
hsa-miR-1228-3p	30.148172	up	1.51E-10
hsa-miR-6069	27.87234	up	2.56E-10
hsa-miR-6800-3p	27.269981	up	2.59E-09
hsa-miR-1238-3p	27.049736	up	6.62E-10
hsa-miR-6737-3p	25.080816	up	1.15E-10
hsa-miR-4730	24.819586	up	1.78E-14
hsa-miR-6716-3p	24.539305	up	8.48E-14
hsa-miR-6508-5p	23.8303	up	5.85E-09
hsa-miR-4433a-5p	20.12776	up	1.30E-10
hsa-miR-451b	19.966757	up	2.79E-08
hsa-miR-5010-3p	19.232067	up	1.62E-08
hsa-miR-191-3p	19.044302	up	1.38E-08
hsa-miR-1234-3p	16.4659	up	1.02E-07
hsa-miR-3162-3p	16.097244	up	9.02E-11
hsa-miR-6797-3p	15.228248	up	3.81E-09
hsa-miR-1281	14.348885	up	1.99E-08
hsa-miR-4455	12.991922	up	3.94E-07
hsa-miR-4665-3p	11.097143	up	4.46E-07
hsa-miR-1825	11.092906	up	1.47E-06
hsa-miR-940	10.92399	up	6.13E-08
hsa-miR-574-5p	10.731104	up	4.42E-07
hsa-miR-1304-3p	9.837506	up	6.04E-05
hsa-miR-6851-3p	9.263779	up	2.47E-05
hsa-miR-4649-3p	9.22571	up	9.86E-07
hsa-miR-6751-3p	8.612906	up	7.93E-05
hsa-miR-4313	7.8738523	up	2.13E-04
hsa-miR-8485	6.791654	up	9.09E-05
hsa-miR-4725-5p	6.5970798	up	4.73E-04
hsa-miR-6870-3p	6.535795	up	4.09E-04
hsa-miR-32-3p	5.6060896	up	3.70E-04
hsa-miR-6813-3p	5.284001	up	9.57E-04
hsa-miR-7114-3p	5.0547495	up	5.93E-04
hsa-miR-4769-3p	4.8945293	up	5.45E-04
hsa-miR-4284	4.627779	up	0.017311715
hsa-miR-6834-3p	3.8669906	up	0.005754791
hsa-miR-3149	3.4039087	up	0.006676147
hsa-miR-1290	3.2591238	up	8.33E-04
hsa-miR-6861-3p	2.8124409	up	0.010480452
hsa-miR-483-3p	2.767849	up	0.036811158
hsa-miR-6515-3p	2.735245	up	0.01791451
hsa-miR-1246	2.6975517	up	1.28E-05

Table S5. Differentially expressed miRNAs were analyzed in plasma rectal cancer patients versus healthy controls. The threshold value for upregulated and downregulated genes was a fold change ≥1.5 and *p*-value ≤0.05.

Systematic_Name	FC (abs)	Regulation	*p*

hsa-miR-642b-3p	-17.698948	down	1.47E-06
hsa-miR-4741	-17.438139	down	7.05E-08
hsa-miR-8072	-16.954582	down	6.86E-07
hsa-miR-195-5p	-16.870594	down	4.58E-09
hsa-miR-134-5p	-15.255262	down	5.22E-08
hsa-miR-1227-5p	-14.240981	down	2.99E-08
hsa-miR-7108-5p	-14.102511	down	6.90E-08
hsa-miR-6728-5p	-14.047849	down	3.49E-09
hsa-miR-6850-5p	-13.927513	down	0.001009182
hsa-miR-142-5p	-13.260134	down	2.65E-05
hsa-miR-192-5p	-13.208113	down	7.88E-08
hsa-miR-4443	-12.575873	down	1.72E-04
hsa-miR-6791-5p	-12.162286	down	6.91E-05
hsa-miR-96-5p	-12.051468	down	1.45E-07
hsa-miR-374a-5p	-11.6988325	down	3.30E-06
hsa-miR-575	-11.611312	down	3.78E-09
hsa-miR-642a-3p	-11.417184	down	0.002989296
hsa-miR-425-5p	-11.215559	down	1.19E-04
hsa-miR-215-5p	-10.99455	down	3.25E-10
hsa-miR-6068	-10.839542	down	4.42E-05
hsa-miR-1202	-10.496189	down	7.10E-04
hsa-miR-3663-3p	-10.39162	down	7.32E-04
hsa-miR-186-5p	-10.336495	down	1.31E-07
hsa-miR-151b	-10.250122	down	6.31E-07
hsa-miR-4530	-10.217537	down	0.001127996
hsa-miR-4739	-9.975182	down	1.35E-06
hsa-miR-5787	-9.938442	down	0.001432164
hsa-miR-660-5p	-9.838545	down	4.02E-05
hsa-miR-144-3p	-9.739871	down	9.79E-05
hsa-miR-4270	-9.561591	down	0.001424078
hsa-miR-363-3p	-9.393585	down	2.50E-04
hsa-miR-30b-5p	-9.281387	down	2.96E-05
hsa-miR-371b-5p	-9.27924	down	1.44E-04
hsa-miR-126-5p	-9.248106	down	3.47E-06
hsa-miR-4788	-9.179674	down	1.21E-08
hsa-miR-6789-5p	-9.112293	down	9.14E-10
hsa-miR-1225-5p	-9.099518	down	0.002235186
hsa-miR-6749-5p	-9.080338	down	0.002189587
hsa-miR-4728-5p	-8.935863	down	2.09E-08
hsa-miR-6727-5p	-8.881601	down	0.002929077
hsa-miR-6786-5p	-8.806383	down	6.19E-08
hsa-miR-4463	-8.784044	down	7.57E-07
hsa-miR-5703	-8.719135	down	0.007174332
hsa-miR-3138	-8.674742	down	7.74E-09
hsa-miR-4534	-8.667723	down	6.05E-04
hsa-miR-148a-3p	-8.302392	down	3.57E-05
hsa-miR-331-3p	-8.24382	down	2.51E-08
hsa-miR-1207-5p	-8.105125	down	0.00282465
hsa-miR-18a-5p	-8.014106	down	3.27E-05
hsa-miR-26b-5p	-8.005609	down	0.00283016
hsa-miR-3135b	-7.968773	down	2.83E-04
hsa-miR-630	-7.815078	down	0.01052535
hsa-miR-638	-7.79626	down	0.004056337
hsa-miR-3195	-7.7535725	down	3.36E-09
hsa-miR-5006-5p	-7.744547	down	1.52E-09
hsa-miR-6763-5p	-7.740162	down	3.02E-07
hsa-miR-939-5p	-7.729882	down	1.52E-08
hsa-miR-374b-5p	-7.622873	down	2.56E-08
hsa-miR-6085	-7.592694	down	8.26E-06
hsa-miR-4763-3p	-7.4306207	down	0.00547572
hsa-miR-30d-5p	-7.341276	down	0.002968174
hsa-miR-8063	-7.2326727	down	2.23E-07
hsa-miR-301a-3p	-7.193112	down	9.35E-06
hsa-miR-4634	-6.9415817	down	2.55E-04
hsa-miR-3652	-6.9143744	down	3.32E-11

hsa-miR-128-3p	-6.899596	down	1.37E-11
hsa-miR-30c-5p	-6.8739023	down	1.23E-06
hsa-miR-572	-6.800901	down	1.10E-06
hsa-miR-328-5p	-6.7855115	down	0.009398649
hsa-miR-148b-3p	-6.721095	down	7.72E-06
hsa-miR-1471	-6.668908	down	1.20E-07
hsa-miR-4442	-6.579573	down	4.75E-05
hsa-miR-340-5p	-6.5751534	down	3.13E-11
hsa-miR-718	-6.5650353	down	2.40E-04
hsa-miR-6775-5p	-6.5413365	down	0.002351017
hsa-miR-5585-3p	-6.506056	down	5.27E-07
hsa-miR-188-5p	-6.5043764	down	2.47E-13
hsa-miR-4651	-6.5019174	down	3.69E-15
hsa-miR-27b-3p	-6.4907494	down	3.24E-04
hsa-miR-1915-3p	-6.462355	down	0.003776004
hsa-miR-6724-5p	-6.436633	down	0.001846714
hsa-miR-4484	-6.413649	down	1.69E-10
hsa-miR-1183	-6.3106236	down	2.49E-08
hsa-miR-4466	-6.267067	down	0.004932348
hsa-miR-3679-5p	-6.215233	down	0.003274665
hsa-miR-342-3p	-6.2028785	down	1.21E-04
hsa-miR-23b-3p	-6.16741	down	1.04E-04
hsa-let-7d-3p	-6.1399474	down	4.92E-06
hsa-miR-4499	-6.088096	down	1.92E-06
hsa-miR-3648	-6.080401	down	1.11E-11
hsa-miR-26a-5p	-6.0503063	down	0.008451654
hsa-miR-1268b	-6.016851	down	1.30E-06
hsa-miR-3188	-5.9901266	down	7.56E-08
hsa-miR-6893-5p	-5.9056025	down	9.92E-13
hsa-miR-151a-3p	-5.877762	down	4.19E-06
hsa-miR-6752-5p	-5.8430524	down	0.001357136
hsa-miR-3196	-5.8197455	down	2.34E-06
hsa-miR-454-3p	-5.793686	down	1.20E-08
hsa-miR-7-5p	-5.74208	down	3.30E-05
hsa-miR-5739	-5.6902986	down	6.16E-04
hsa-miR-4787-5p	-5.620427	down	0.001065759
hsa-miR-4721	-5.580764	down	2.46E-15
hsa-miR-6829-5p	-5.5240984	down	5.12E-11
hsa-miR-4327	-5.5043283	down	3.11E-07
hsa-miR-130b-3p	-5.5023494	down	1.87E-04
hsa-miR-5195-3p	-5.411947	down	2.08E-07
hsa-miR-150-3p	-5.4075403	down	1.71E-07
hsa-miR-548q	-5.400439	down	4.48E-11
hsa-miR-125a-5p	-5.347823	down	6.49E-08
hsa-miR-4271	-5.295589	down	2.69E-08
hsa-miR-6812-5p	-5.2812304	down	5.94E-05
hsa-miR-6869-5p	-5.2776175	down	0.030215487
hsa-miR-6124	-5.27679	down	0.008558582
hsa-miR-3940-5p	-5.2688847	down	8.79E-05
hsa-miR-1229-5p	-5.0768313	down	0.009309867
hsa-miR-183-5p	-5.051148	down	4.69E-06
hsa-miR-3667-5p	-5.045067	down	2.40E-06
hsa-miR-937-5p	-5.0117774	down	1.21E-05
hsa-miR-6780b-5p	-4.9605746	down	1.66E-04
hsa-miR-671-5p	-4.9200864	down	1.76E-07
hsa-miR-4459	-4.9040217	down	0.006731615
hsa-miR-6879-5p	-4.844546	down	3.84E-04
hsa-miR-7641	-4.825062	down	0.00478969
hsa-miR-3194-5p	-4.818217	down	3.57E-15
hsa-miR-6126	-4.8168564	down	5.04E-12
hsa-miR-6785-5p	-4.7778673	down	0.008942611
hsa-miR-7150	-4.759289	down	0.035733562
hsa-miR-6875-5p	-4.7458205	down	0.003345262
hsa-miR-8064	-4.740493	down	8.50E-09
hsa-miR-181a-5p	-4.721017	down	2.63E-04

hsa-miR-3656	-4.7097487	down	0.016498437
hsa-miR-6127	-4.6833744	down	0.002618671
hsa-miR-324-5p	-4.6811886	down	7.56E-07
hsa-miR-590-5p	-4.665492	down	0.003303035
hsa-miR-6858-5p	-4.665387	down	2.15E-11
hsa-miR-4745-5p	-4.6496744	down	4.92E-12
hsa-miR-30e-5p	-4.638218	down	0.009462235
hsa-miR-1914-3p	-4.6320653	down	1.23E-19
hsa-miR-27a-3p	-4.588405	down	0.008616984
hsa-miR-140-5p	-4.5777035	down	1.23E-04
hsa-miR-4687-3p	-4.534422	down	0.039401792
hsa-miR-6756-5p	-4.5099926	down	1.11E-05
hsa-miR-345-3p	-4.501176	down	1.12E-09
hsa-miR-135a-3p	-4.4671125	down	3.49E-05
hsa-miR-6794-5p	-4.4656944	down	4.16E-04
hsa-miR-19a-3p	-4.4238	down	0.013169073
hsa-miR-18b-5p	-4.3512444	down	7.64E-05
hsa-miR-199a-5p	-4.3336153	down	0.005794328
hsa-miR-1249-5p	-4.32165	down	4.57E-09
hsa-miR-584-5p	-4.3162	down	1.27E-08
hsa-miR-3937	-4.290447	down	1.14E-09
hsa-miR-2276-3p	-4.2245	down	5.19E-09
hsa-miR-10b-5p	-4.153251	down	2.57E-07
hsa-miR-532-5p	-4.112886	down	4.63E-06
hsa-miR-7845-5p	-4.1087456	down	7.89E-11
hsa-miR-210-3p	-4.0628104	down	3.96E-04
hsa-miR-320a	-4.047538	down	0.019352725
hsa-miR-6800-5p	-3.992329	down	0.014373192
hsa-miR-7106-5p	-3.9794679	down	7.69E-04
hsa-miR-762	-3.962642	down	0.007135358
hsa-miR-376a-3p	-3.7817614	down	2.87E-05
hsa-miR-6086	-3.7762034	down	1.37E-06
hsa-miR-484	-3.7688918	down	0.004909118
hsa-miR-146a-5p	-3.7541137	down	0.012143113
hsa-miR-30a-5p	-3.6294549	down	1.35E-04
hsa-miR-320b	-3.612441	down	0.038985133
hsa-miR-378i	-3.6068876	down	1.95E-05
hsa-miR-335-5p	-3.5446124	down	8.81E-08
hsa-miR-197-5p	-3.5375495	down	0.013917983
hsa-miR-146b-5p	-3.5189464	down	7.29E-07
hsa-miR-2861	-3.4493215	down	0.014101845
hsa-miR-198	-3.4323826	down	2.04E-04
hsa-miR-424-5p	-3.3857808	down	0.001470275
hsa-miR-106b-5p	-3.3380468	down	0.009031892
hsa-miR-194-5p	-3.3178694	down	5.41E-07
hsa-miR-2392	-3.2619255	down	0.020649794
hsa-miR-5001-5p	-3.2328167	down	0.027117865
hsa-miR-4707-3p	-3.2270503	down	8.88E-08
hsa-miR-338-3p	-3.1831071	down	4.55E-05
hsa-miR-7704	-3.1803765	down	0.031313114
hsa-miR-17-3p	-3.1761377	down	0.004250472
hsa-miR-652-5p	-3.1549861	down	2.76E-14
hsa-miR-3141	-3.1356945	down	0.015659565
hsa-miR-4778-5p	-3.1171834	down	0.008131617
hsa-miR-3665	-3.0952525	down	0.041360788
hsa-miR-3610	-3.0642486	down	2.63E-05
hsa-miR-557	-3.060216	down	8.06E-08
hsa-miR-98-5p	-2.997998	down	0.002315295
hsa-miR-498	-2.995054	down	1.46E-07
hsa-miR-125b-5p	-2.988829	down	1.35E-05
hsa-miR-4800-5p	-2.9831712	down	0.016404362
hsa-miR-6790-5p	-2.9542782	down	1.16E-16
hsa-miR-221-3p	-2.9496	down	0.03162353
hsa-miR-6075	-2.9324594	down	1.11E-13
hsa-miR-378a-3p	-2.9291847	down	7.36E-06

hsa-miR-34a-5p	-2.927698	down	1.11E-06
hsa-miR-623	-2.924685	down	3.93E-07
hsa-miR-6820-5p	-2.9161675	down	2.88E-07
hsa-miR-365a-3p	-2.8485901	down	4.35E-08
hsa-miR-6730-5p	-2.8261933	down	3.08E-11
hsa-miR-486-3p	-2.7457156	down	2.06E-04
hsa-miR-3158-5p	-2.69376	down	4.57E-14
hsa-miR-10a-5p	-2.6498177	down	6.24E-06
hsa-miR-4429	-2.6189446	down	9.07E-05
hsa-miR-15b-5p	-2.5906572	down	7.21E-04
hsa-miR-361-3p	-2.5072474	down	1.52E-07
hsa-miR-29b-3p	-2.4845538	down	0.036073163
hsa-miR-222-3p	-2.4703245	down	0.01965298
hsa-miR-497-5p	-2.4539943	down	1.01E-07
hsa-miR-4481	-2.3321133	down	1.57E-09
hsa-miR-100-5p	-2.313144	down	3.58E-06
hsa-miR-3960	-2.2312152	down	0.001537408
hsa-let-7g-5p	-2.1676872	down	0.022192793
hsa-miR-19b-3p	-2.1611707	down	0.008539365
hsa-miR-139-3p	-2.0268016	down	2.31E-09
hsa-miR-6779-5p	-2.014083	down	0.019162955
hsa-miR-20b-5p	-1.9796937	down	0.010479953
hsa-miR-629-5p	-1.8917848	down	8.69E-06
hsa-miR-15a-5p	-1.8419242	down	0.001552703
hsa-miR-20a-5p	-1.61354	down	0.02919387
hsa-miR-29c-3p	-1.5472715	down	0.016652932
hsa-miR-6508-5p	44.94544	up	6.03E-12
hsa-miR-6800-3p	42.488583	up	2.66E-11
hsa-miR-451b	40.17751	up	7.25E-12
hsa-miR-1228-3p	38.353195	up	7.91E-11
hsa-miR-1238-3p	34.811417	up	4.82E-11
hsa-miR-4730	34.731014	up	1.34E-11
hsa-miR-6069	34.25672	up	2.10E-10
hsa-miR-6716-3p	33.76955	up	3.28E-11
hsa-miR-5010-3p	31.934872	up	1.68E-10
hsa-miR-191-3p	31.08507	up	8.26E-11
hsa-miR-1234-3p	30.505447	up	1.99E-10
hsa-miR-6737-3p	29.85762	up	2.50E-10
hsa-miR-4455	28.234776	up	2.65E-09
hsa-miR-4433a-5p	24.42316	up	8.13E-10
hsa-miR-1281	24.314016	up	3.42E-09
hsa-miR-3162-3p	23.158672	up	9.54E-11
hsa-miR-574-5p	22.594849	up	7.79E-10
hsa-miR-6797-3p	21.93045	up	1.34E-09
hsa-miR-1825	20.717909	up	5.68E-09
hsa-miR-6751-3p	19.310602	up	1.27E-06
hsa-miR-4665-3p	15.921819	up	6.51E-09
hsa-miR-6813-3p	15.844627	up	1.86E-07
hsa-miR-8485	15.750625	up	2.02E-07
hsa-miR-940	15.240282	up	1.13E-08
hsa-miR-6851-3p	14.757802	up	2.81E-07
hsa-miR-3149	14.740364	up	4.66E-06
hsa-miR-4313	14.07879	up	1.94E-05
hsa-miR-32-3p	13.450186	up	1.32E-06
hsa-miR-6834-3p	11.825755	up	2.01E-05
hsa-miR-4769-3p	10.706	up	1.11E-05
hsa-miR-4725-5p	10.448515	up	2.11E-04
hsa-miR-4649-3p	10.081085	up	4.32E-05
hsa-miR-483-3p	10.046119	up	1.28E-05
hsa-miR-1304-3p	9.078493	up	1.94E-04
hsa-miR-6870-3p	9.036732	up	3.87E-05
hsa-miR-6515-3p	8.509136	up	1.23E-04
hsa-miR-7114-3p	7.2395573	up	2.39E-04
hsa-miR-4290	6.2279506	up	0.001038709
hsa-miR-6861-3p	5.682808	up	0.001392494

hsa-miR-1290	5.4939084	up	2.33E-04
hsa-miR-6739-5p	5.270004	up	1.19E-04
hsa-miR-1246	4.7447033	up	1.65E-06
hsa-miR-4284	4.4530253	up	0.008798013
hsa-miR-4749-3p	3.8275385	up	0.003176451
hsa-miR-1273g-3p	3.442122	up	0.001952466
hsa-miR-6819-3p	3.2139478	up	0.031974927
hsa-miR-548c-3p	2.5497031	up	0.018937416
hsa-miR-6766-3p	2.530503	up	0.026634071
hsa-miR-1224-3p	2.112045	up	0.014022677

Table S6. Differentially expressed miRNAs were analyzed in plasma colorectal cancer patients versus healthy controls. The threshold value for upregulated and downregulated genes was a fold change ≥1.5 and *p*-value ≤0.05.

Systematic_Name	FC (abs)	Regulation	*p*
hsa-miR-195-5p	-18.817083	down	6.57E-17
hsa-miR-96-5p	-16.276388	down	2.54E-18
hsa-miR-4741	-16.049957	down	7.21E-10
hsa-miR-363-3p	-15.419561	down	9.54E-07
hsa-miR-642b-3p	-14.913443	down	1.99E-07
hsa-miR-134-5p	-13.088778	down	2.41E-09
hsa-miR-192-5p	-12.807455	down	9.69E-11
hsa-miR-374a-5p	-11.839737	down	1.20E-07
hsa-miR-660-5p	-11.375271	down	2.03E-07
hsa-miR-6068	-11.091023	down	4.17E-06
hsa-miR-6728-5p	-10.82846	down	6.99E-10
hsa-miR-425-5p	-10.548464	down	3.01E-05
hsa-miR-142-5p	-10.319482	down	3.09E-05
hsa-miR-30b-5p	-10.255639	down	8.51E-07
hsa-miR-151a-5p	-10.212569	down	9.21E-08
hsa-miR-215-5p	-10.07147	down	1.39E-14
hsa-miR-126-5p	-10.044064	down	1.81E-09
hsa-miR-1227-5p	-9.993745	down	1.30E-08
hsa-miR-151b	-9.945766	down	1.97E-09
hsa-miR-301a-3p	-9.706256	down	4.01E-09
hsa-miR-6791-5p	-9.649806	down	1.66E-04
hsa-miR-18a-5p	-9.370826	down	1.17E-07
hsa-miR-148a-3p	-9.130793	down	2.65E-06
hsa-miR-186-5p	-9.109111	down	1.06E-08
hsa-miR-642a-3p	-9.106543	down	0.003948664
hsa-miR-371b-5p	-9.037191	down	9.12E-05
hsa-miR-4534	-8.788887	down	2.58E-04
hsa-miR-8072	-8.701094	down	6.08E-05
hsa-miR-4728-5p	-8.697706	down	4.50E-17
hsa-miR-7108-5p	-8.57047	down	6.65E-06
hsa-miR-4443	-8.552064	down	2.72E-04
hsa-miR-4530	-8.535406	down	0.001600046
hsa-miR-5787	-8.417286	down	0.001988932
hsa-miR-575	-8.3517	down	2.95E-09
hsa-miR-27b-3p	-8.132425	down	1.01E-05
hsa-miR-6850-5p	-8.059588	down	0.003773439
hsa-miR-26b-5p	-8.017587	down	3.80E-04
hsa-miR-4788	-7.9594493	down	2.28E-11
hsa-miR-5739	-7.921967	down	2.98E-05
hsa-miR-4634	-7.9133234	down	1.81E-05
hsa-miR-3135b	-7.6615214	down	1.44E-06
hsa-miR-331-3p	-7.6080284	down	7.00E-11
hsa-miR-4739	-7.5708833	down	1.01E-06
hsa-miR-590-5p	-7.522014	down	1.49E-05
hsa-miR-148b-3p	-7.456761	down	3.13E-08
hsa-miR-3138	-7.438351	down	1.89E-10
hsa-miR-6789-5p	-7.3977494	down	5.61E-11
hsa-miR-6085	-7.253052	down	3.15E-06

hsa-miR-7-5p	-7.178249	down	1.89E-10
hsa-miR-6749-5p	-7.141727	down	0.004406519
hsa-miR-374b-5p	-7.032165	down	1.71E-11
hsa-miR-30c-5p	-7.006509	down	6.52E-09
hsa-miR-4442	-6.8117385	down	5.20E-06
hsa-miR-1202	-6.8062596	down	0.002387004
hsa-miR-5703	-6.7925115	down	0.013649669
hsa-miR-6786-5p	-6.572998	down	8.41E-07
hsa-miR-630	-6.5669317	down	0.0160374
hsa-miR-3195	-6.4262743	down	1.01E-10
hsa-miR-3663-3p	-6.298948	down	0.002452866
hsa-miR-128-3p	-6.2682095	down	3.41E-15
hsa-miR-183-5p	-6.2638874	down	7.16E-14
hsa-miR-718	-6.1670556	down	1.01E-04
hsa-miR-939-5p	-6.1567235	down	5.99E-09
hsa-miR-572	-6.143451	down	1.10E-08
hsa-miR-638	-6.0846047	down	0.007724057
hsa-miR-3679-5p	-6.0663605	down	0.001795867
hsa-miR-4270	-6.0591116	down	0.004546233
hsa-miR-5585-3p	-5.979514	down	1.48E-09
hsa-miR-23b-3p	-5.931212	down	9.71E-06
hsa-miR-6752-5p	-5.9222207	down	2.56E-04
hsa-miR-4463	-5.82371	down	4.17E-05
hsa-miR-1225-5p	-5.7989573	down	0.008056652
hsa-miR-18b-5p	-5.7592382	down	6.40E-08
hsa-miR-1207-5p	-5.6783795	down	0.007325315
hsa-miR-1471	-5.667467	down	8.00E-09
hsa-miR-5006-5p	-5.6112366	down	1.51E-08
hsa-miR-1915-3p	-5.538143	down	0.008179117
hsa-miR-6763-5p	-5.5240726	down	6.74E-07
hsa-miR-8063	-5.5046577	down	8.57E-08
hsa-miR-188-5p	-5.4637866	down	2.37E-17
hsa-miR-3940-5p	-5.455228	down	1.55E-05
hsa-miR-6869-5p	-5.454521	down	0.020603845
hsa-miR-342-3p	-5.4326143	down	5.38E-04
hsa-miR-6727-5p	-5.409926	down	0.008533103
hsa-miR-4651	-5.385709	down	6.31E-18
hsa-miR-4459	-5.376338	down	0.013653612
hsa-miR-454-3p	-5.3466787	down	1.26E-15
hsa-miR-130b-3p	-5.339442	down	7.91E-05
hsa-miR-3188	-5.2901726	down	4.60E-09
hsa-miR-1183	-5.266278	down	1.14E-09
hsa-miR-6724-5p	-5.1408005	down	0.004485659
hsa-miR-3652	-5.0073633	down	1.92E-08
hsa-miR-4484	-4.959791	down	3.91E-10
hsa-miR-340-5p	-4.958847	down	4.22E-11
hsa-miR-140-5p	-4.9532743	down	4.18E-06
hsa-miR-3196	-4.940166	down	2.50E-06
hsa-miR-6780b-5p	-4.9353385	down	5.51E-07
hsa-miR-98-5p	-4.8731217	down	6.04E-09
hsa-miR-4466	-4.7887235	down	0.012988478
hsa-miR-199a-5p	-4.7726145	down	0.001234816
hsa-miR-150-3p	-4.7547145	down	2.23E-09
hsa-miR-4763-3p	-4.709969	down	0.021727553
hsa-miR-6127	-4.6826367	down	0.001969452
hsa-miR-6893-5p	-4.6469536	down	6.91E-14
hsa-miR-548q	-4.6302056	down	1.93E-14
hsa-miR-125a-5p	-4.6221566	down	6.85E-11
hsa-miR-6829-5p	-4.5898786	down	8.70E-14
hsa-let-7d-3p	-4.5557494	down	9.23E-05
hsa-miR-146a-5p	-4.545061	down	0.002496355
hsa-miR-6800-5p	-4.518268	down	0.016603379
hsa-miR-4271	-4.5100737	down	3.17E-09
hsa-miR-6875-5p	-4.438074	down	2.08E-05
hsa-miR-324-5p	-4.4222517	down	4.73E-09

hsa-miR-532-5p	-4.402987	down	7.14E-12
hsa-miR-5195-3p	-4.365667	down	2.60E-07
hsa-miR-7641	-4.343594	down	0.001507537
hsa-miR-144-5p	-4.3235927	down	1.09E-05
hsa-miR-4499	-4.2906756	down	2.32E-05
hsa-miR-3648	-4.2686577	down	2.90E-14
hsa-miR-671-5p	-4.2634115	down	9.26E-09
hsa-miR-4327	-4.2425714	down	1.16E-06
hsa-miR-6812-5p	-4.196215	down	5.73E-04
hsa-miR-424-5p	-4.1724997	down	3.34E-05
hsa-miR-10b-5p	-4.1393824	down	2.41E-10
hsa-miR-19a-3p	-4.128786	down	0.004145203
hsa-miR-6775-5p	-4.12333	down	0.020697972
hsa-miR-210-3p	-4.1130724	down	3.12E-06
hsa-miR-3667-5p	-4.092896	down	2.88E-06
hsa-miR-6794-5p	-4.0526137	down	8.61E-04
hsa-miR-1268b	-4.0522842	down	2.28E-05
hsa-miR-484	-3.9926472	down	9.02E-04
hsa-miR-937-5p	-3.9748037	down	4.57E-05
hsa-miR-197-5p	-3.968531	down	0.016957127
hsa-miR-6124	-3.9442096	down	0.021335324
hsa-miR-181a-5p	-3.9428988	down	2.54E-04
hsa-miR-376c-3p	-3.9367557	down	3.87E-08
hsa-miR-6879-5p	-3.9321866	down	0.001633666
hsa-miR-345-3p	-3.8832836	down	1.82E-12
hsa-miR-3194-5p	-3.8685493	down	7.96E-14
hsa-miR-4721	-3.8504777	down	9.05E-12
hsa-miR-3937	-3.8199637	down	8.74E-17
hsa-miR-8064	-3.730656	down	2.57E-08
hsa-miR-6858-5p	-3.7172866	down	9.27E-12
hsa-miR-2276-3p	-3.7057345	down	1.40E-11
hsa-miR-376a-3p	-3.6979203	down	2.42E-08
hsa-miR-4745-5p	-3.6714063	down	6.50E-13
hsa-miR-135a-3p	-3.6567793	down	2.59E-05
hsa-miR-3141	-3.607383	down	0.007044534
hsa-miR-584-5p	-3.6054554	down	2.43E-08
hsa-miR-320a	-3.6046314	down	0.026258538
hsa-miR-335-5p	-3.5905132	down	1.61E-14
hsa-miR-1914-3p	-3.5813653	down	2.12E-22
hsa-miR-6126	-3.5677655	down	7.63E-11
hsa-miR-3656	-3.5512922	down	0.03379125
hsa-miR-762	-3.5477028	down	0.007860391
hsa-miR-7847-3p	-3.5377665	down	0.035498
hsa-miR-6831-5p	-3.5358574	down	0.01728924
hsa-miR-27a-3p	-3.510689	down	0.022960031
hsa-miR-486-3p	-3.454302	down	2.87E-11
hsa-miR-17-3p	-3.43685	down	2.66E-04
hsa-miR-146b-5p	-3.3099542	down	5.29E-10
hsa-miR-1249-5p	-3.2941897	down	4.93E-09
hsa-miR-2861	-3.293926	down	0.022118533
hsa-miR-6756-5p	-3.28809	down	1.50E-04
hsa-miR-29b-3p	-3.2052658	down	0.00967639
hsa-let-7e-5p	-3.197785	down	2.01E-05
hsa-miR-3610	-3.1922328	down	4.32E-12
hsa-miR-378i	-3.1648498	down	3.88E-05
hsa-miR-16-2-3p	-3.1548777	down	0.00455271
hsa-miR-7845-5p	-3.1460974	down	4.05E-10
hsa-miR-6086	-3.1291914	down	1.11E-06
hsa-miR-377-3p	-3.126262	down	4.52E-14
hsa-miR-194-5p	-3.1255758	down	1.32E-09
hsa-miR-500a-3p	-3.111047	down	3.56E-11
hsa-miR-320b	-3.1086102	down	0.039215714
hsa-miR-1185-1-3p	-3.0617254	down	8.89E-09
hsa-miR-6833-5p	-3.0580056	down	1.71E-04
hsa-miR-4778-5p	-3.056587	down	0.003259339

hsa-miR-221-3p	-3.0081816	down	0.025633406
hsa-miR-7106-5p	-3.0031521	down	0.004926544
hsa-miR-4476	-3.001603	down	6.84E-11
hsa-miR-1226-5p	-2.9840605	down	7.26E-15
hsa-let-7g-5p	-2.9649425	down	0.002565142
hsa-let-7f-5p	-2.9450636	down	0.036886238
hsa-let-7c-5p	-2.8964581	down	0.02764962
hsa-miR-106b-5p	-2.880392	down	0.009247184
hsa-miR-6723-5p	-2.8754272	down	2.79E-09
hsa-miR-4707-3p	-2.8653567	down	4.33E-12
hsa-miR-362-3p	-2.8307793	down	1.02E-31
hsa-miR-20b-5p	-2.8220801	down	0.001286004
hsa-miR-8087	-2.7844002	down	1.39E-12
hsa-miR-10a-5p	-2.7692397	down	2.13E-11
hsa-miR-198	-2.7467237	down	0.001696119
hsa-miR-338-3p	-2.7454107	down	8.60E-05
hsa-miR-125b-5p	-2.7241395	down	4.89E-06
hsa-miR-30a-5p	-2.7066905	down	0.008074927
hsa-miR-6819-5p	-2.6976192	down	2.18E-06
hsa-miR-6076	-2.697427	down	6.83E-06
hsa-miR-6889-5p	-2.6741095	down	1.08E-13
hsa-miR-4472	-2.6384926	down	1.33E-06
hsa-miR-498	-2.6317935	down	3.11E-08
hsa-miR-4689	-2.6007855	down	3.07E-10
hsa-miR-3945	-2.558	down	1.44E-09
hsa-miR-652-5p	-2.5550196	down	1.01E-14
hsa-miR-328-3p	-2.4896476	down	0.003918324
hsa-miR-4800-5p	-2.4697614	down	0.032500193
hsa-miR-32-5p	-2.4687228	down	1.24E-23
hsa-miR-34a-5p	-2.4650748	down	5.55E-06
hsa-miR-365a-3p	-2.4522202	down	1.33E-09
hsa-miR-6730-5p	-2.427055	down	7.16E-16
hsa-miR-936	-2.422345	down	1.15E-12
hsa-miR-557	-2.4170825	down	1.49E-05
hsa-miR-5196-5p	-2.414294	down	2.66E-09
hsa-miR-378a-3p	-2.4078681	down	9.16E-05
hsa-miR-361-3p	-2.404694	down	6.46E-13
hsa-miR-550a-3p	-2.401832	down	4.54E-08
hsa-miR-6738-5p	-2.391808	down	2.43E-10
hsa-miR-625-5p	-2.3885949	down	3.16E-08
hsa-miR-6806-5p	-2.357881	down	3.26E-11
hsa-miR-623	-2.3486319	down	1.44E-05
hsa-miR-1273f	-2.3472059	down	1.15E-07
hsa-miR-3911	-2.333487	down	3.70E-04
hsa-miR-6075	-2.3249643	down	1.57E-10
hsa-miR-550a-3-5p	-2.3247764	down	8.07E-14
hsa-miR-500b-5p	-2.3205512	down	1.26E-19
hsa-miR-3158-5p	-2.317846	down	1.14E-16
hsa-miR-6820-5p	-2.3088505	down	1.80E-05
hsa-miR-502-3p	-2.2820218	down	2.08E-08
hsa-miR-497-5p	-2.2775123	down	7.66E-10
hsa-miR-4429	-2.2772121	down	7.68E-05
hsa-miR-6790-5p	-2.275993	down	3.22E-07
hsa-miR-28-5p	-2.2643461	down	8.66E-12
hsa-miR-4318	-2.2464552	down	4.09E-10
hsa-miR-505-3p	-2.236741	down	5.58E-12
hsa-miR-1224-5p	-2.2265306	down	4.08E-08
hsa-miR-3156-5p	-2.2008436	down	6.54E-14
hsa-miR-6073	-2.1952627	down	1.40E-04
hsa-miR-19b-3p	-2.182291	down	0.001249115
hsa-miR-222-3p	-2.1678562	down	0.033816654
hsa-miR-6778-5p	-2.1623857	down	3.60E-07
hsa-miR-15b-3p	-2.1525402	down	5.76E-23
hsa-miR-4314	-2.1511378	down	1.93E-07
hsa-miR-4701-3p	-2.1483965	down	0.001433074

hsa-miR-29c-5p	-2.143977	down	3.15E-10
hsa-miR-196b-5p	-2.1385567	down	1.47E-20
hsa-miR-100-5p	-2.1351373	down	1.85E-09
hsa-miR-15a-5p	-2.1227117	down	4.08E-05
hsa-miR-495-3p	-2.1154683	down	6.24E-09
hsa-miR-22-5p	-2.0690458	down	3.23E-13
hsa-miR-624-5p	-2.0602736	down	7.98E-22
hsa-miR-4695-5p	-2.031634	down	9.59E-05
hsa-miR-6840-3p	-2.0093098	down	7.40E-10
hsa-let-7i-5p	-2.0085988	down	5.63E-04
hsa-miR-25-3p	-2.0036635	down	0.002725539
hsa-miR-4481	-1.997419	down	5.80E-14
hsa-miR-20a-5p	-1.992616	down	0.001618002
hsa-miR-6757-5p	-1.9623383	down	1.86E-10
hsa-miR-4673	-1.9251508	down	1.26E-09
hsa-miR-4734	-1.9112371	down	1.40E-08
hsa-miR-6802-5p	-1.8871756	down	4.04E-09
hsa-miR-99a-5p	-1.8753809	down	6.92E-08
hsa-miR-601	-1.8691542	down	1.76E-07
hsa-miR-1185-2-3p	-1.8633478	down	5.18E-08
hsa-miR-145-5p	-1.8623804	down	3.66E-04
hsa-miR-136-5p	-1.856118	down	9.59E-04
hsa-miR-139-3p	-1.8543397	down	8.61E-13
hsa-miR-5685	-1.8530449	down	9.10E-06
hsa-miR-629-5p	-1.8488889	down	8.91E-07
hsa-miR-6768-5p	-1.8224642	down	7.51E-11
hsa-miR-3960	-1.8120283	down	0.009938992
hsa-miR-7846-3p	-1.7743001	down	2.63E-04
hsa-miR-6809-5p	-1.7526833	down	0.029643845
hsa-miR-378d	-1.7186202	down	3.17E-09
hsa-miR-4496	-1.7063788	down	5.32E-07
hsa-miR-187-5p	-1.6826344	down	4.76E-07
hsa-miR-30e-3p	-1.6719811	down	4.26E-05
hsa-miR-8089	-1.6492912	down	1.69E-05
hsa-miR-3202	-1.6320455	down	0.001248828
hsa-miR-1343-5p	-1.6278356	down	5.91E-05
hsa-miR-4746-3p	-1.627519	down	1.04E-05
hsa-miR-4787-3p	-1.6266427	down	6.14E-04
hsa-miR-4767	-1.6191354	down	5.44E-09
hsa-miR-3137	-1.6032785	down	0.011920111
hsa-miR-4322	-1.6000853	down	5.57E-06
hsa-miR-6129	-1.5939972	down	3.26E-05
hsa-miR-8073	-1.5894003	down	1.87E-04
hsa-miR-196a-5p	-1.5715226	down	5.73E-07
hsa-miR-6784-5p	-1.5477245	down	2.89E-04
hsa-miR-654-3p	-1.5470378	down	9.51E-05
hsa-miR-487b-3p	-1.543488	down	0.002326863
hsa-miR-4253	-1.5357696	down	1.65E-04
hsa-miR-3923	-1.5347615	down	2.28E-06
hsa-miR-3198	-1.5345788	down	5.26E-04
hsa-miR-4317	-1.5343933	down	9.93E-09
hsa-miR-133b	-1.5296113	down	7.33E-05
hsa-miR-6071	-1.5261133	down	6.58E-05
hsa-miR-513a-5p	-1.5219287	down	0.011546293
hsa-miR-224-5p	-1.5217549	down	5.49E-06
hsa-miR-6741-5p	-1.5131516	down	0.026460359
hsa-miR-1228-3p	32.27718	up	3.42E-12
hsa-miR-4730	30.422903	up	2.83E-17
hsa-miR-6716-3p	29.729202	up	1.68E-16
hsa-miR-6800-3p	29.503895	up	9.18E-11
hsa-miR-6508-5p	28.357412	up	8.31E-11
hsa-miR-6069	28.025434	up	1.00E-11
hsa-miR-1238-3p	27.997011	up	1.77E-11
hsa-miR-451b	27.348436	up	6.54E-11
hsa-miR-6737-3p	24.871214	up	6.56E-12

hsa-miR-5010-3p	23.709328	up	1.11E-10
hsa-miR-191-3p	21.585037	up	3.30E-10
hsa-miR-4433a-5p	20.750298	up	6.51E-12
hsa-miR-1234-3p	20.488674	up	1.48E-09
hsa-miR-3162-3p	19.78715	up	3.22E-13
hsa-miR-1281	19.084553	up	2.54E-10
hsa-miR-4455	18.082542	up	4.14E-09
hsa-miR-6797-3p	18.079847	up	6.06E-11
hsa-miR-574-5p	15.281876	up	3.38E-09
hsa-miR-1825	14.909924	up	2.33E-08
hsa-miR-4665-3p	14.3801985	up	6.29E-09
hsa-miR-940	14.21207	up	5.06E-10
hsa-miR-6751-3p	11.924637	up	6.65E-06
hsa-miR-6851-3p	10.274067	up	4.08E-06
hsa-miR-8485	10.240803	up	1.65E-06
hsa-miR-1304-3p	9.692887	up	4.43E-05
hsa-miR-4313	9.627616	up	4.77E-05
hsa-miR-4649-3p	8.847954	up	2.86E-06
hsa-miR-4725-5p	7.8299713	up	1.93E-04
hsa-miR-6813-3p	7.5761566	up	3.55E-05
hsa-miR-32-3p	7.5108023	up	3.72E-05
hsa-miR-6870-3p	7.2413974	up	9.60E-05
hsa-miR-4769-3p	6.9003167	up	5.20E-05
hsa-miR-6834-3p	5.953099	up	3.99E-04
hsa-miR-3149	5.6869464	up	4.85E-04
hsa-miR-7114-3p	5.6578975	up	2.53E-04
hsa-miR-4446-5p	5.199757	up	0.001480785
hsa-miR-4284	4.7353096	up	0.012844542
hsa-miR-1250-3p	4.440918	up	0.001619956
hsa-miR-483-3p	4.3843226	up	0.003350844
hsa-miR-6514-3p	4.2128963	up	0.008658452
hsa-miR-6515-3p	4.108173	up	0.003259649
hsa-miR-1290	3.9771118	up	2.92E-04
hsa-miR-4485-5p	3.619606	up	0.035324756
hsa-miR-6861-3p	3.578525	up	0.003532203
hsa-miR-6889-3p	3.514845	up	0.008078669
hsa-miR-1246	3.4087684	up	1.19E-06
hsa-miR-6760-3p	3.3645709	up	0.005375398
hsa-miR-3653-5p	3.3227017	up	0.001715339
hsa-miR-491-3p	3.0987763	up	0.002226062
hsa-miR-933	2.9197109	up	0.004580116
hsa-miR-1273g-3p	2.7311556	up	0.006227011
hsa-miR-6798-3p	2.6818657	up	0.012433725
hsa-miR-4290	2.638919	up	0.037525527
hsa-miR-6880-3p	2.1112335	up	0.024522016
hsa-miR-4749-3p	2.060287	up	0.020719018
hsa-miR-6778-3p	1.9191736	up	0.013868927
hsa-miR-3613-3p	1.8628482	up	0.040795192
hsa-miR-34b-3p	1.8161502	up	0.025234645
hsa-miR-1224-3p	1.7235461	up	0.035547186
hsa-miR-6805-5p	1.5911901	up	0.030013535

Table S7. Differentially expressed miRNAs were analyzed in plasma chemotreated colorectal cancer patients versus untreated colorectal cancer patients. The threshold value for upregulated and downregulated genes was a fold change ≥1.5 and *p*-value ≤0.05.

Systematic_Name	FC (abs)	Regulation	*p*
hsa-miR-574-3p	-4.707971	down	0.007044553
hsa-miR-6088	-4.2144275	down	0.003552679
hsa-miR-4455	-3.8249462	down	0.011860294
hsa-miR-6125	-3.7428992	down	0.004982985
hsa-miR-6740-5p	-3.725341	down	0.039324444
hsa-miR-5010-3p	-3.2811484	down	0.021567397
hsa-miR-32-3p	-2.9936733	down	0.03313809

hsa-miR-6165	-2.942446	down	0.039437376
hsa-miR-1290	-2.670601	down	0.04024074
hsa-miR-4730	-2.2495148	down	0.04175604
hsa-miR-6090	-2.2029164	down	0.04453954
hsa-miR-6089	-2.135594	down	0.005230125
hsa-miR-4281	-1.7625313	down	0.025285373
hsa-miR-3960	-1.7338134	down	0.029885752
hsa-miR-18a-5p	4.6639194	up	0.001662072
hsa-miR-151a-3p	4.4174294	up	0.001037019
hsa-let-7d-5p	4.233113	up	0.010206928
hsa-miR-103a-3p	4.15344	up	0.013946099
hsa-miR-26b-5p	3.9169278	up	0.021480478
hsa-miR-30e-5p	3.7584565	up	0.008996997
hsa-miR-26a-5p	3.6667118	up	0.029952237
hsa-miR-199a-5p	3.595766	up	0.020379977
hsa-let-7f-5p	3.4678154	up	0.025064226
hsa-miR-23b-3p	3.2887168	up	0.012643079
hsa-miR-144-3p	3.201038	up	0.038210653
hsa-miR-17-5p	3.1981778	up	0.009441602
hsa-miR-29b-3p	3.0401678	up	0.018676355
hsa-miR-30b-5p	3.0304313	up	0.030820938
hsa-miR-19a-3p	2.9517112	up	0.024809351
hsa-miR-18b-5p	2.9143608	up	0.004686276
hsa-miR-30c-5p	2.9088764	up	0.006498599
hsa-miR-374a-5p	2.8217683	up	0.035894904
hsa-miR-148b-3p	2.607857	up	0.020650163
hsa-miR-328-3p	2.5699759	up	0.020868253
hsa-miR-181a-5p	2.543037	up	0.04186078
hsa-miR-151b	2.5332196	up	0.028695533
hsa-miR-93-5p	2.397497	up	0.027022392
hsa-miR-374b-5p	2.371192	up	0.01429387
hsa-miR-34a-5p	2.2413929	up	0.004126904
hsa-miR-106b-5p	2.2385614	up	0.041558307
hsa-miR-324-3p	2.2074447	up	0.041203555
hsa-miR-210-3p	2.2065916	up	0.046626933
hsa-miR-20b-5p	2.1872258	up	0.017583672
hsa-miR-361-5p	2.138619	up	0.031487927
hsa-miR-584-5p	2.1347876	up	0.008861961
hsa-miR-96-5p	2.101457	up	0.011548122
hsa-miR-331-3p	1.9966366	up	0.037425395
hsa-miR-126-3p	1.9670222	up	0.018098893
hsa-miR-195-5p	1.8922216	up	0.042993996
hsa-miR-20a-5p	1.8627847	up	0.004240367
hsa-miR-215-5p	1.8601036	up	0.04361076
hsa-miR-532-5p	1.8593402	up	0.01576892
hsa-miR-19b-3p	1.8193731	up	0.013355121
hsa-miR-183-5p	1.7478797	up	0.021310113
hsa-miR-652-3p	1.7190313	up	0.044040028
hsa-miR-25-3p	1.6944741	up	0.02510068
hsa-miR-10b-5p	1.671827	up	0.046438057
hsa-miR-194-5p	1.6507075	up	0.034387264
hsa-miR-486-3p	1.6445782	up	0.01744914
hsa-miR-128-3p	1.642851	up	0.033004418
hsa-miR-16-5p	1.6003872	up	0.030637063
hsa-miR-21-5p	1.596436	up	0.013982079
hsa-miR-629-5p	1.5757582	up	0.005779247

Table S8. Differentially expressed miRNAs were analyzed in plasma chemotreated rectal cancer patients versus untreated rectal cancer patients. The threshold value for upregulated and downregulated genes was a fold change ≥1.5 and *p*-value ≤0.05.

Systematic_Name	FC (abs)	Regulation	*p*
hsa-miR-4455	-10.197052	down	0.002471766
hsa-miR-6740-5p	-8.901676	down	0.010032822

hsa-miR-574-3p	-8.493112	down	0.010908118
hsa-miR-3149	-7.9205465	down	0.003406616
hsa-miR-32-3p	-6.936792	down	0.003502164
hsa-miR-5010-3p	-6.359786	down	0.014490048
hsa-miR-6834-3p	-5.377581	down	0.030148871
hsa-miR-6739-5p	-4.8212924	down	0.007088768
hsa-miR-451b	-3.7644587	down	0.036476903
hsa-miR-4644	-3.4251516	down	0.01447643
hsa-miR-26a-5p	8.998737	up	0.007867808
hsa-let-7d-5p	8.37634	up	0.003975279
hsa-miR-103a-3p	7.846926	up	0.010587392
hsa-miR-26b-5p	7.004991	up	0.010193903
hsa-miR-30e-5p	5.6959457	up	0.007506873
hsa-miR-144-3p	5.5556955	up	0.005611194
hsa-miR-425-5p	5.441935	up	0.03630426
hsa-let-7f-5p	5.226171	up	0.025616886
hsa-miR-18a-5p	5.0511136	up	0.027735166
hsa-miR-19a-3p	4.863106	up	0.016271124
hsa-miR-151a-3p	3.9572845	up	0.025120396
hsa-miR-30c-5p	3.79025	up	0.0304551
hsa-miR-29b-3p	3.787197	up	0.00868337
hsa-miR-23a-3p	3.3684056	up	0.019133346
hsa-miR-106b-5p	3.2983665	up	0.020186564
hsa-miR-34a-5p	3.097207	up	0.011985444
hsa-miR-130a-3p	2.9837472	up	0.030885218
hsa-miR-17-5p	2.7596178	up	0.004184775
hsa-let-7a-5p	2.6237125	up	0.033776604
hsa-miR-15b-5p	2.5663142	up	0.001061199
hsa-miR-126-3p	2.4664588	up	0.008646518
hsa-miR-215-5p	2.4504719	up	0.049638417
hsa-miR-19b-3p	2.4384582	up	0.004268087
hsa-miR-142-3p	2.3890023	up	0.040102866
hsa-let-7g-5p	2.2943914	up	0.023863621
hsa-miR-93-5p	2.229338	up	0.014466448
hsa-miR-20b-5p	2.2016134	up	0.005933316
hsa-miR-185-5p	2.0949988	up	0.049758106
hsa-miR-101-3p	2.0491412	up	0.0189567
hsa-miR-29c-3p	2.016969	up	8.56E-04
hsa-miR-21-5p	1.9557222	up	0.008012681
hsa-miR-107	1.9434578	up	0.037316296
hsa-miR-629-5p	1.8806	up	0.015035483
hsa-miR-20a-5p	1.8104604	up	0.011932527
hsa-miR-4306	1.796793	up	0.024286147
hsa-miR-22-3p	1.742627	up	0.007275642
hsa-miR-16-5p	1.6699768	up	0.012902103
hsa-miR-25-3p	1.6550758	up	0.042343654
hsa-miR-15a-5p	1.6123587	up	0.004987674
hsa-let-7i-5p	1.5934038	up	0.020621313

© 2020 by the authors. Licensee MDPI, Basel, Switzerland. This article is an open access article distributed under the terms and conditions of the Creative Commons Attribution (CC BY) license (http://creativecommons.org/licenses/by/4.0/).
